# Identification of progressive pulmonary fibrosis: consensus findings from a modified Delphi study

**DOI:** 10.1186/s12931-024-03070-z

**Published:** 2024-12-31

**Authors:** Athol U. Wells, Simon L. F. Walsh, Ayodeji Adegunsoye, Vincent Cottin, Sonye K. Danoff, Anand Devaraj, Kevin R. Flaherty, Peter M. George, Kerri A. Johannson, Martin Kolb, Yasuhiro Kondoh, Andrew G. Nicholson, Sara Tomassetti, Elizabeth R. Volkmann, Kevin K. Brown

**Affiliations:** 1 Royal Brompton and Harefield Hospitals, Guy’s and St Thomas’ NHS Foundation Trust, Sydney Street, London, SW3 6NP UK; 2 National Heart and Lung Institute, Imperial College London, London, UK; 3 University of Chicago, Chicago, USA; 4 National Reference Center for Rare Pulmonary Diseases, Louis Pradel Hospital, Claude Bernard University Lyon 1, Lyon, France; 5 Johns Hopkins Medicine, Baltimore, USA; 6 University of Michigan, Ann Arbor, USA; 7 University of Calgary, Calgary, Canada; 8 McMaster University and St. Joseph’s Healthcare, Hamilton, Canada; 9 Tosei General Hospital, Aichi, Japan; 10 Florence University, Florence, Italy; 11 University of California, David Geffen School of Medicine, Los Angeles, USA; 12 National Jewish Health, Denver, USA

**Keywords:** Disease progression, Fibrosis, pulmonary, Interstitial lung disease, Monitoring, physiologic, Pulmonary function tests

## Abstract

**Background:**

We sought consensus among practising respiratory physicians on the prediction, identification and monitoring of progression in patients with fibrosing interstitial lung disease (ILD) using a modified Delphi process.

**Methods:**

Following a literature review, statements on the prediction, identification and monitoring of progression of ILD were developed by a panel of physicians with specialist expertise. Practising respiratory physicians were sent a survey asking them to indicate their level of agreement with these statements on a binary scale or 7-point Likert scale (− 3 to 3), or to select answers from a list. Consensus was considered to be achieved if ≥ 70% of respondents selected the same answer, or, for responses on a Likert scale, the median score was ≤ –2 (disagree/not important) or ≥ 2 (agree/important) with an interquartile range ≤ 1. There were three rounds of the survey.

**Results:**

Surveys 1, 2 and 3 were completed by 207, 131 and 94 physicians, respectively, between March 2022 and July 2023. Decline in forced vital capacity (FVC), decline in diffusing capacity of the lungs for carbon monoxide, and increased fibrosis on high-resolution computed tomography (HRCT) were ranked as the most important endpoints for determining progression. Consensus was reached that progression on HRCT or a decline in FVC ≥ 10% from baseline is sufficient to determine progression, and that small declines in multiple endpoints indicates progression. Consensus was reached that a histological pattern of usual interstitial pneumonia (UIP) is a risk factor for progression of ILD, but that a biopsy to look for a UIP pattern should not be performed solely for prognostic reasons. Consensus was not reached on the time period over which progression should be defined. There was consensus that appropriate management of ILD depends on the type of ILD, and that ‘despite adequate management’ or ‘despite usual management’ should be included in the definition of progression.

**Conclusions:**

This modified Delphi process provided consensus statements on the identification of ILD progression that were supported by a broad group of clinicians and may help to inform clinical practice until robust evidence-based guidelines are available.

**Supplementary Information:**

The online version contains supplementary material available at 10.1186/s12931-024-03070-z.

## Background

The term progressive pulmonary fibrosis (PPF) is often used to describe lung fibrosis with clinical evidence of progression in a patient with a fibrosing interstitial lung disease (ILD) other than idiopathic pulmonary fibrosis (IPF) [1]. In clinical trials, PPF has been defined in a variety of ways, generally based on a combination of decline in forced vital capacity (FVC) and/or diffusing capacity of the lung for carbon monoxide (DLco), worsening of respiratory symptoms, and/or worsening of abnormalities on high-resolution computed tomography (HRCT), occurring within a period of six months to two years [2–5]. A clinical practice guideline published by an international group of respiratory societies in 2022 proposed that PPF be defined based on at least two of the following occurring within the past year: worsening of respiratory symptoms, absolute decline in FVC % predicted ≥ 5% or decline in DLco % predicted ≥ 10%, and radiological progression [1]. However, the guideline committee acknowledged that this recommendation should be revisited as new evidence becomes available, and there remains no consensus on how PPF should be defined.

While a number of risk factors for the progression of pulmonary fibrosis have been identified [6–8], the course of disease for an individual patient remains unpredictable. Experts in the field have made proposals for how patients with ILD should be monitored using pulmonary function tests (PFTs), assessment of symptoms, and repeat CTs [9–14] but there are few data to inform these proposals. To provide guidance pending the collection of robust evidence, we sought consensus among a large group of practising respiratory physicians on the prediction, identification and monitoring of progression in patients with fibrosing ILD.

## Methods

A PubMed search was performed for papers published between 1 January 2016 and 20 May 2021 using the following search terms: (Progres*) AND (fibros*) AND (ILD). A total of 149 papers were identified and reviewed. Following the literature review, a set of statements was developed by a panel of physicians with specialist expertise in the diagnosis and management of ILD (AUW, SLFW, VC, SKD, AD, KRF, KAJ, MK, YK, AGN, ST, ERV, KKB). The topics covered by the statements were prediction of the progression of ILD, monitoring of ILD, management of ILD, and identification of PPF in clinical practice.

A group of 405 practising respiratory physicians were identified via an internet search, from the European Respiratory Society (ERS) Diffuse Parenchymal Lung Disease Assembly, and from the American Thoracic Society (ATS) Clinical Problems Assembly. These physicians were sent a survey asking them to indicate their level of agreement with statements on a binary scale or on a 7-point Likert scale (from − 3 [strongly disagree/not at all important] to 3 [strongly agree/very important]), or to select answers from a list. Consensus was considered to be achieved if ≥ 70% of respondents agreed with a statement on a binary scale or selected the same answer from the list provided, or, for responses on a Likert scale, when the median score was ≤ –2 (disagree/not important) or ≥ 2 (agree/important) with an interquartile range (IQR) ≤ 1. There were three rounds of the survey. Statements that reached consensus in the first or second rounds were not repeated. Statements that were regarded as close to consensus were adapted (reworded for clarity) and surveyed in the next round. Statements that did not reach consensus and could not be adapted were excluded.

## Results

Surveys were completed between March 2022 and July 2023. The international guideline on the definition of PPF [1] was published between distribution of the first and second rounds of the survey. The first survey was sent to 405 physicians in 32 countries and completed by 207, of whom 131 completed the second round and 94 completed the third round. The clinical experience of the respondents is summarised in Table S1 in Additional file 1. For survey 1, 98% of respondents were pulmonologists and 86% were working at an academic centre/teaching hospital.

**Fig. 1:**
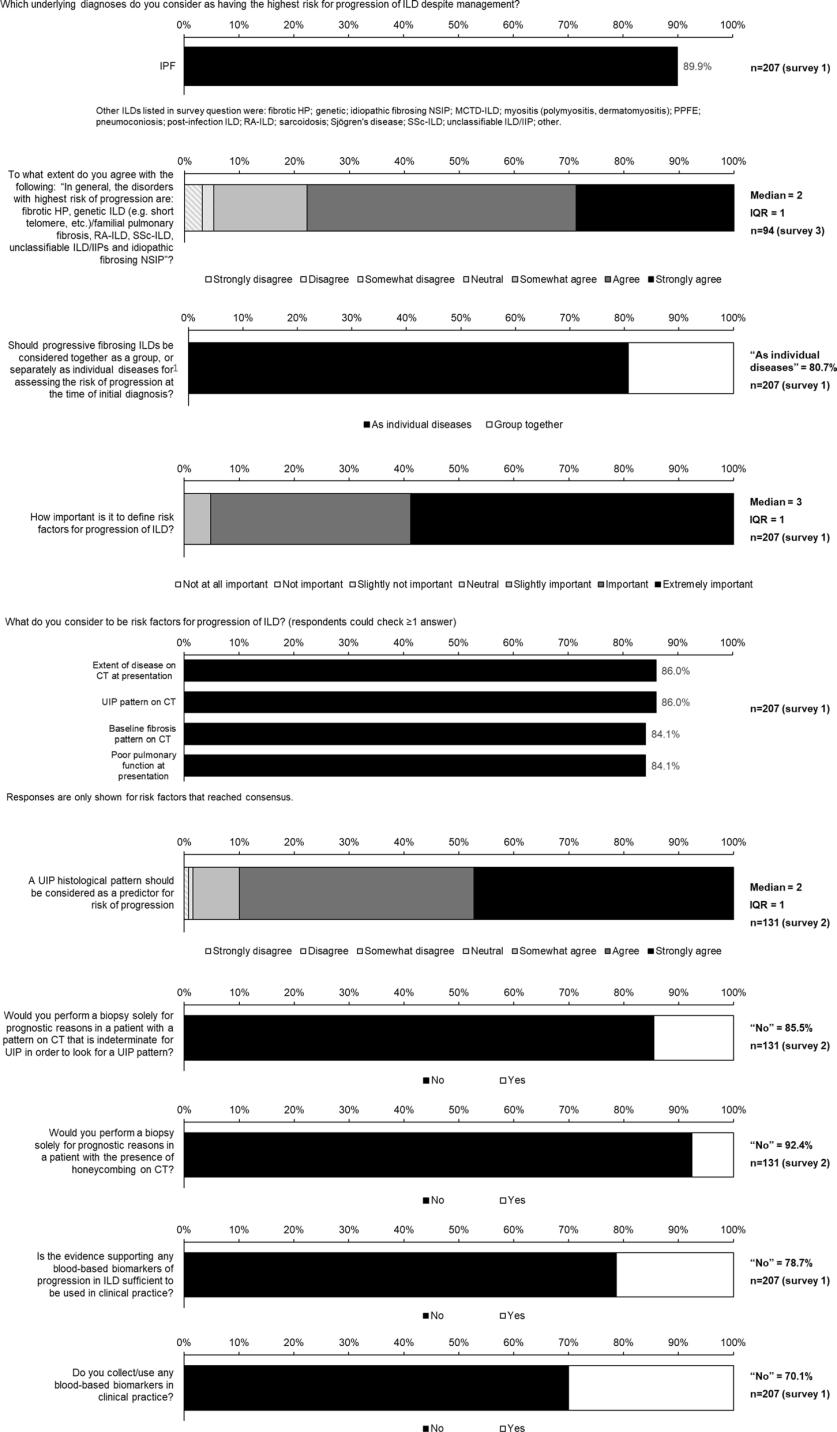
Consensus statements on prediction of progression of ILD

**Fig. 2:**
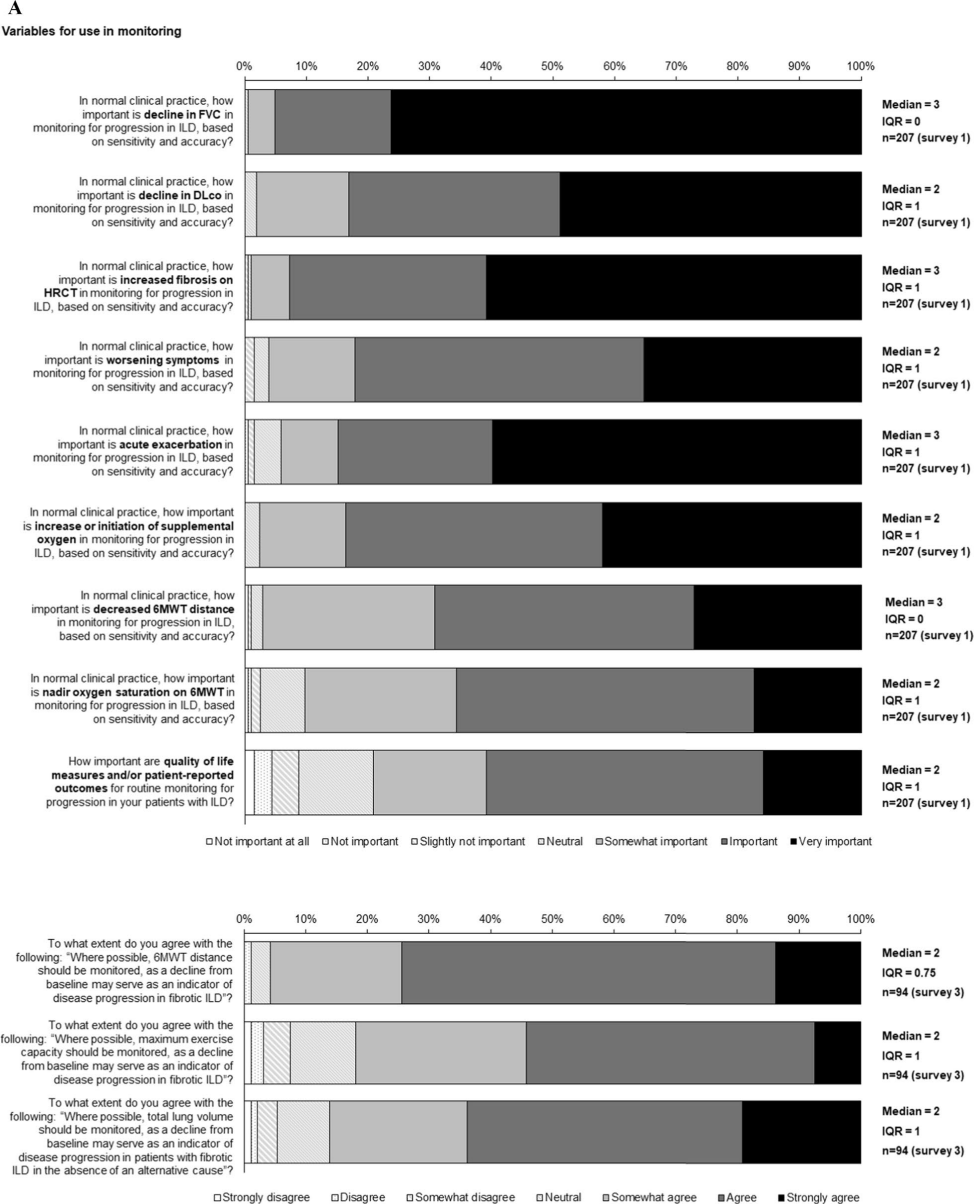

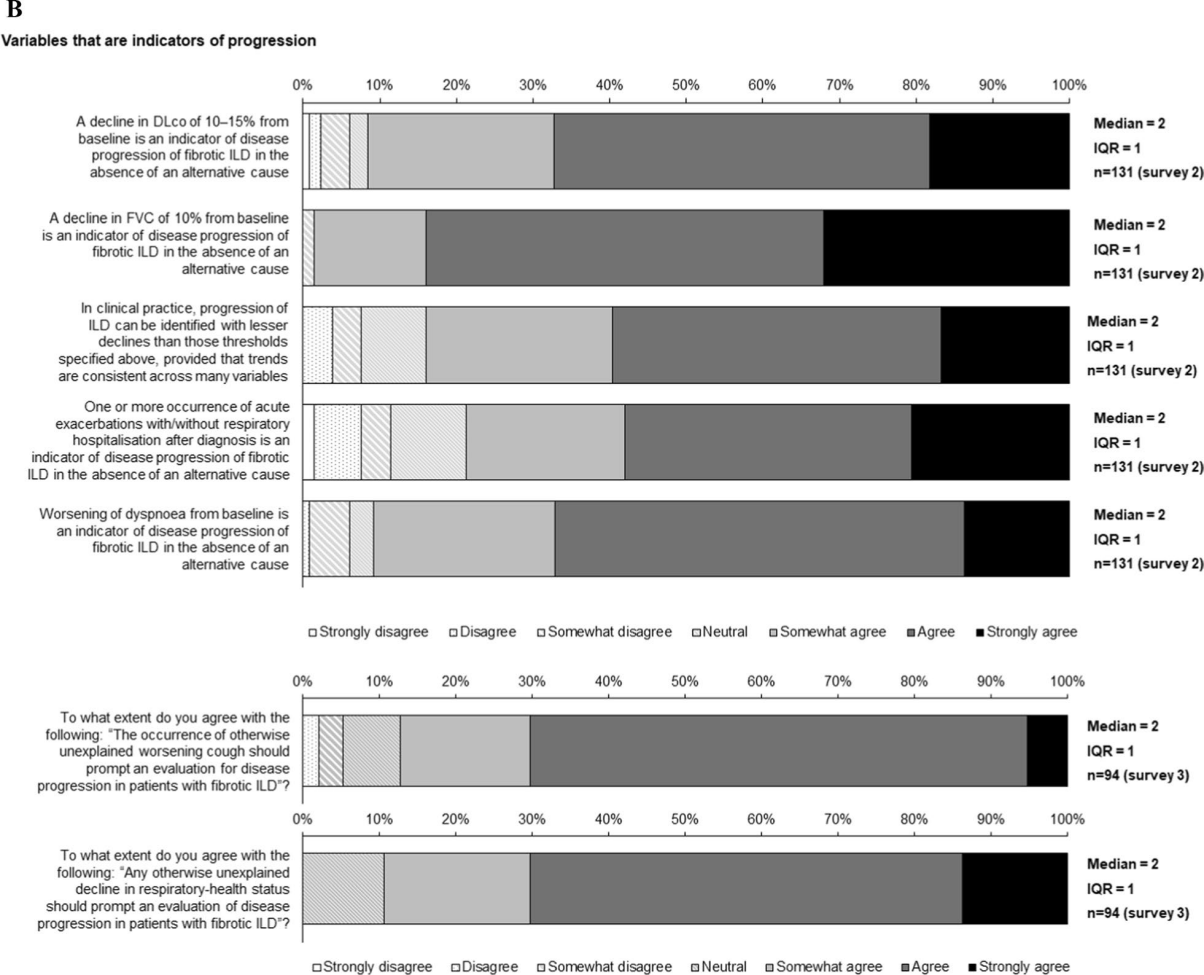

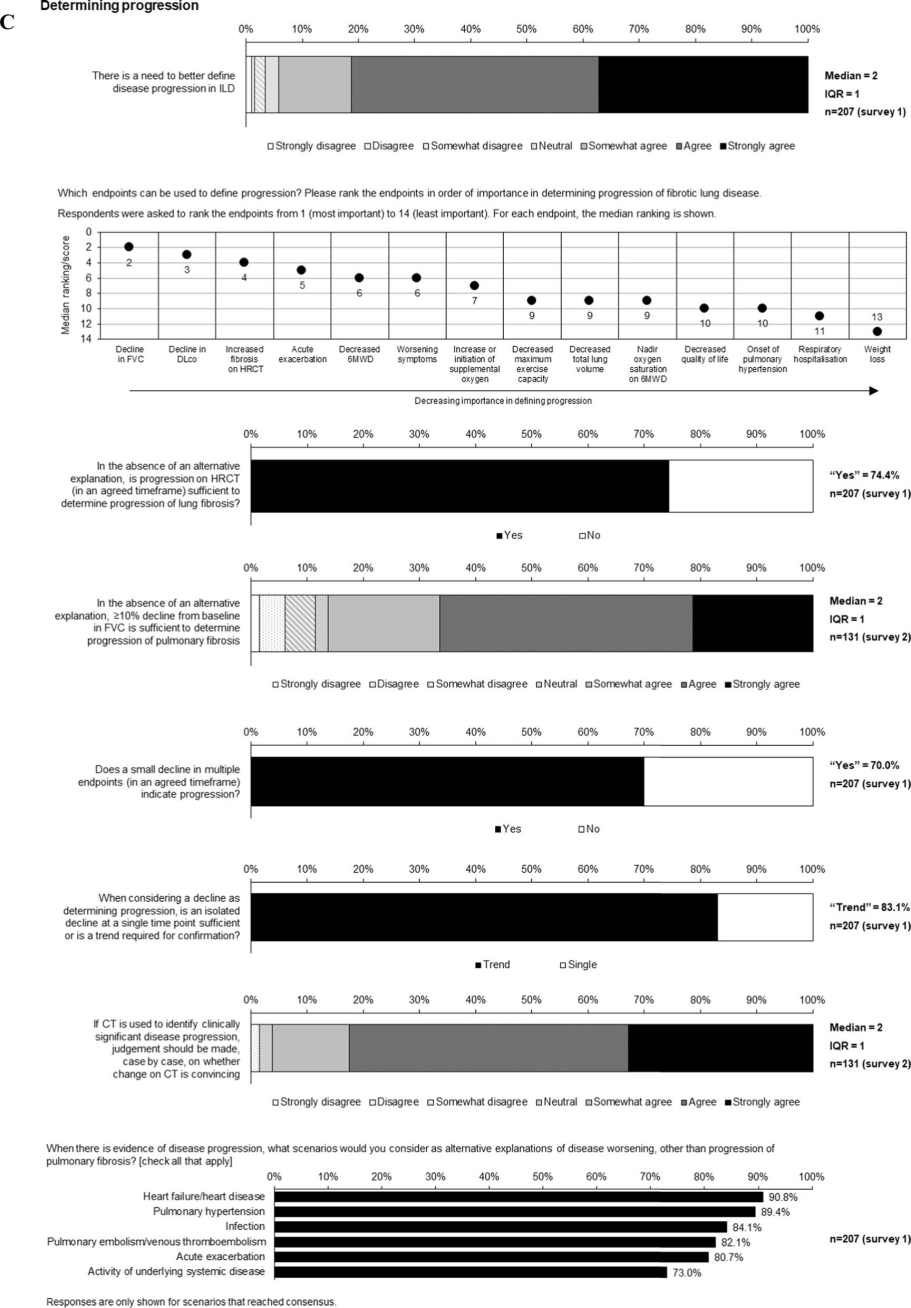
Consensus statements on **A** variables for use in monitoring for progression of ILD, **B** variables that are indicators of progression of ILD and **C** determining progression of ILD

**Fig. 3:**
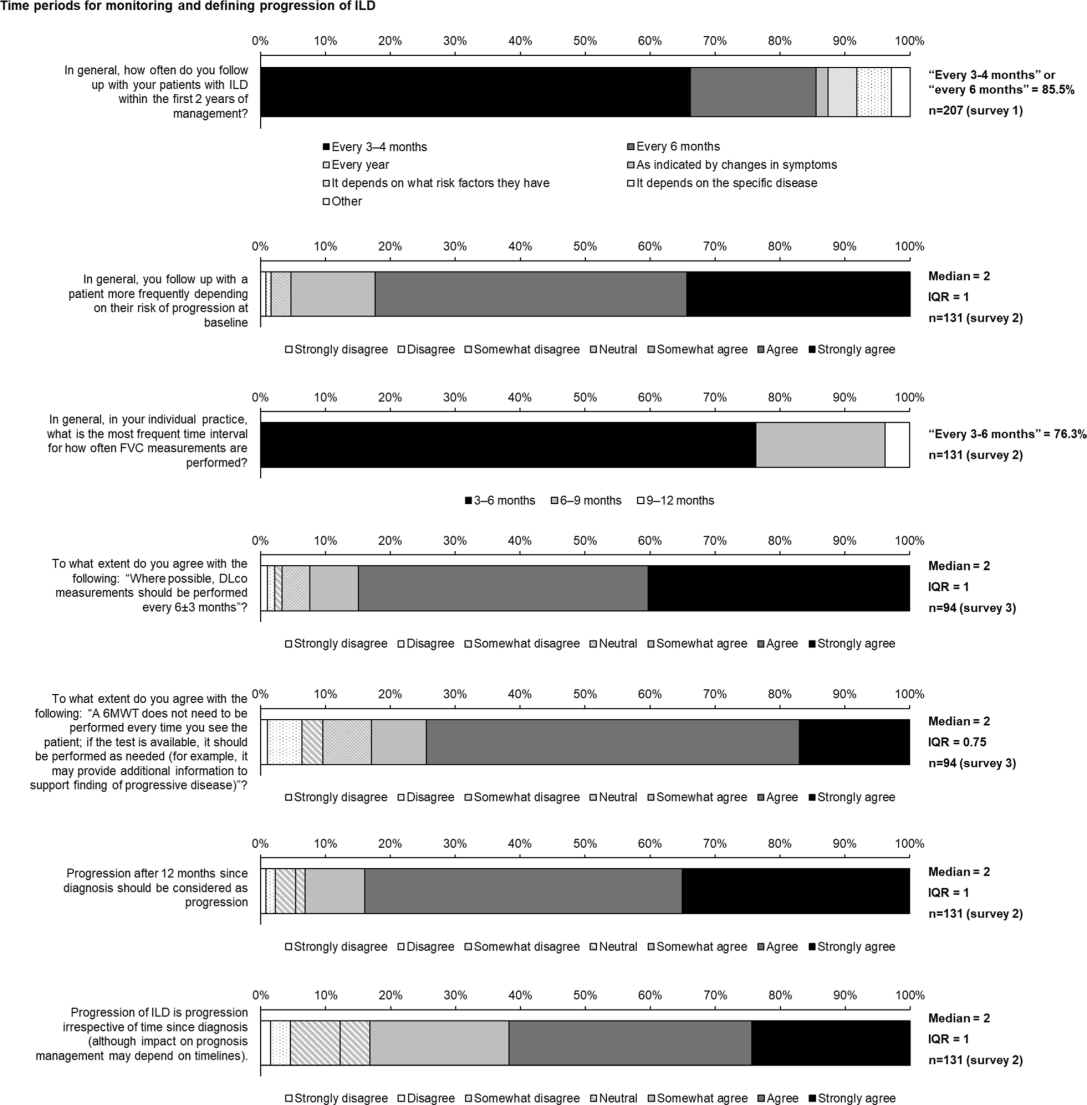
Consensus statements on time periods for monitoring and defining progression of ILD

**Fig. 4:**
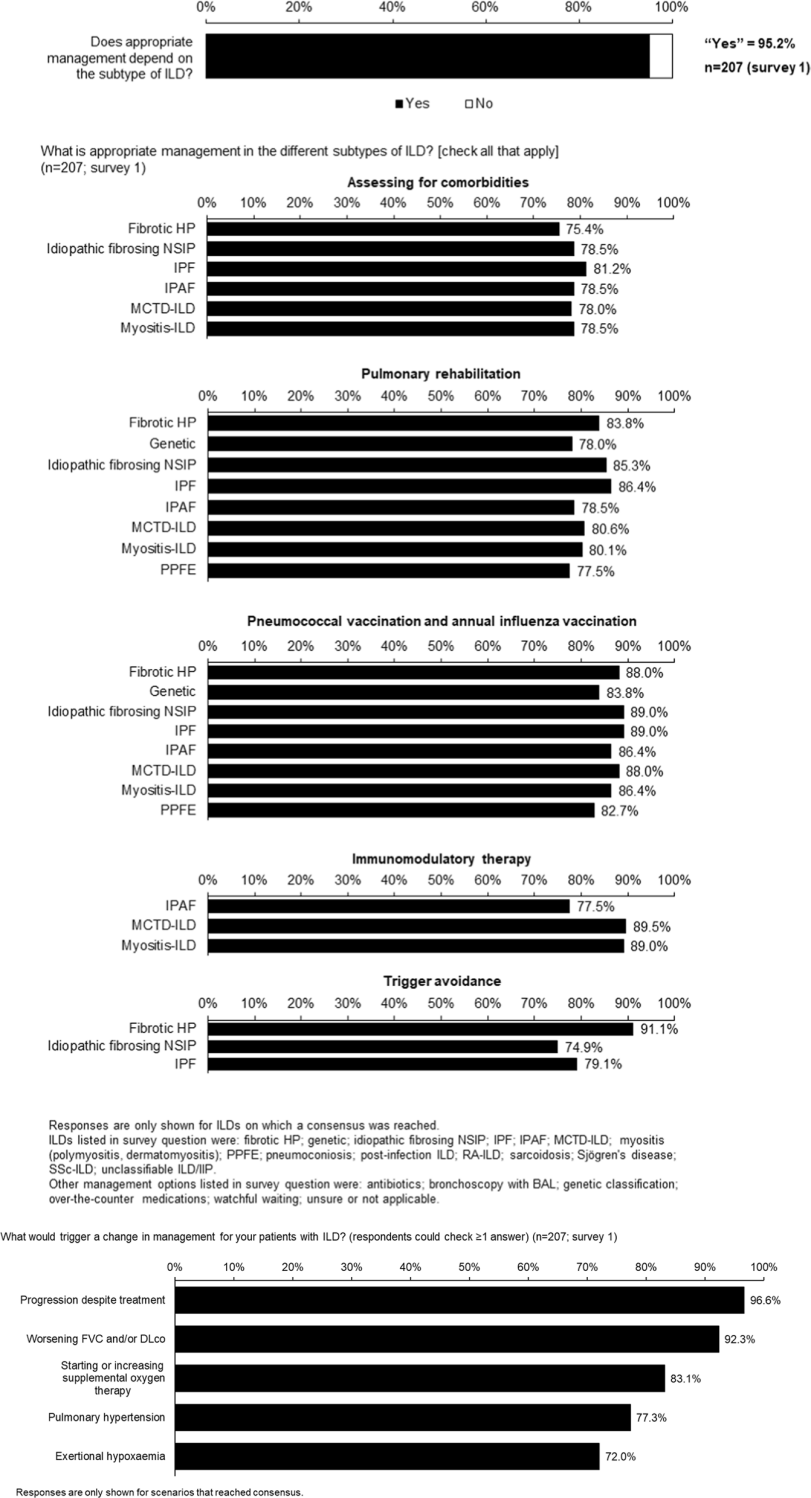
Consensus statements on management of ILD before progression

**Fig. 5:**
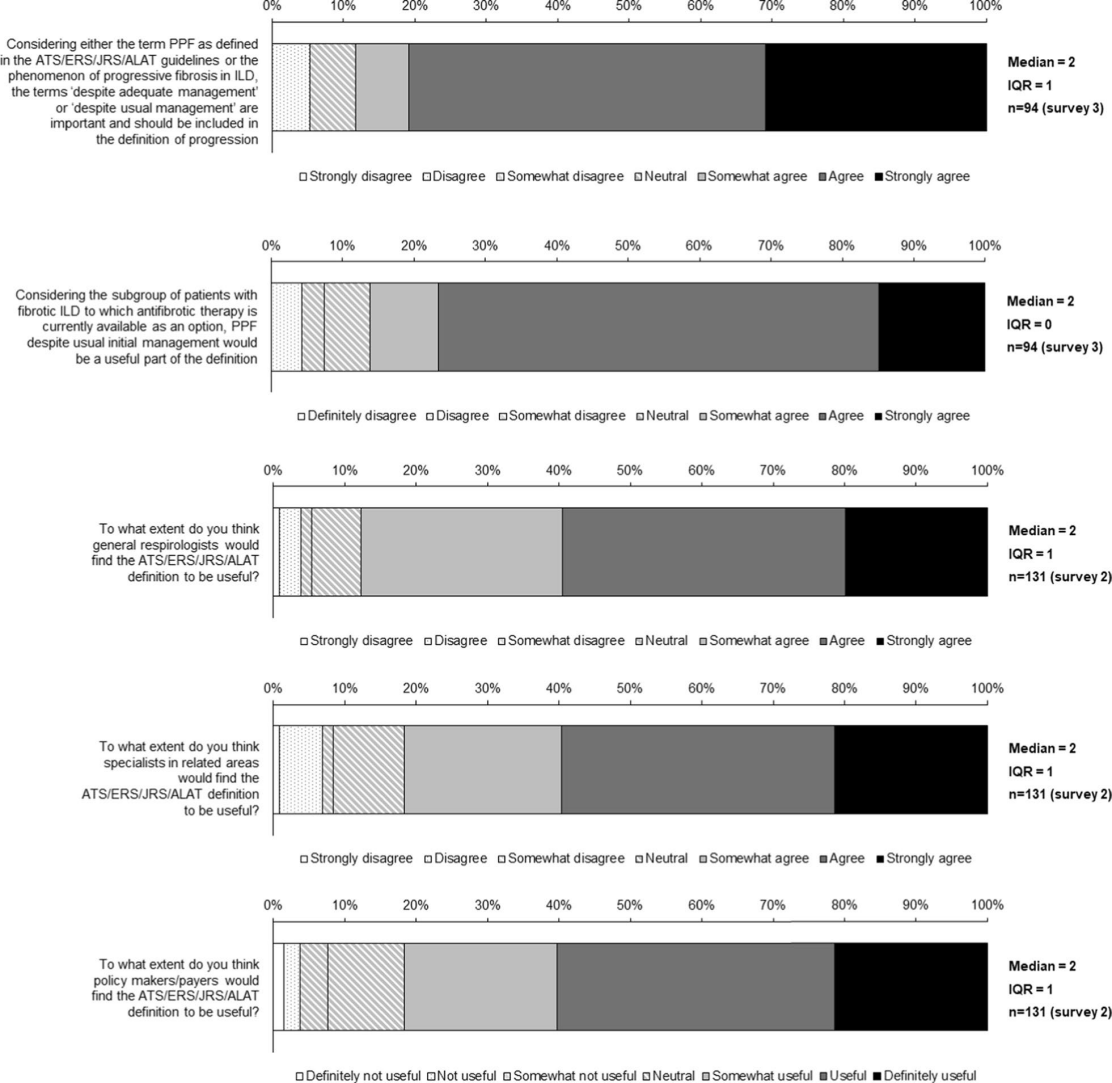
Consensus statements on defining PPF

Statements for which consensus was reached are shown in Figs. 1, 2, 3, 4, 5 and Figs. S1 and S2 in Additional file 1.

### Prediction of progression of ILD

There was consensus that, other than IPF, the ILDs with the highest risk of progression are fibrotic hypersensitivity pneumonitis (HP), genetic ILD/familial pulmonary fibrosis, rheumatoid arthritis-associated ILD (RA-ILD), systemic sclerosis-associated ILD (SSc-ILD), unclassifiable ILD/idiopathic interstitial pneumonias (IIPs) and idiopathic fibrosing non-specific interstitial pneumonia (NSIP) (Fig. 1).

Consensus was reached that it is important to define risk factors for progression of ILD and that these include the extent of disease on computed tomography (CT), a usual interstitial pneumonia (UIP) pattern on CT and poor pulmonary function (Fig. 1; Table S2 in Additional file 1). Consensus on risk factors for progression of individual ILDs are shown in Supplementary Fig. 1. Consensus was reached that while a histological pattern of UIP is a risk factor for progression, a biopsy to look for a UIP pattern would not be performed solely for prognostic reasons (Fig. 1 and Table S2 in Table S2 in Additional file 1). Consensus was not reached on whether bronchoalveolar lavage should be performed for prognostic reasons (Table S2 in Additional file 1). There was consensus that there is insufficient evidence to support the use of any blood-based biomarkers for predicting progression of ILD in clinical practice (Fig. 1).

### Variables for monitoring and defining progression of ILD

Consensus was reached that important variables to monitor progression of ILD include FVC, DLco, fibrosis on HRCT, symptoms, acute exacerbation, initiation or increase of supplemental oxygen, 6-min walk test (6MWT) distance and nadir oxygen saturation on 6MWT (Fig. 2A, supplementary Table 3). In addition, consensus was reached that quality of life measures/patient-reported outcomes are important for routine monitoring (Fig. 2A). There was consensus that 6MWT distance, maximum exercise capacity, or total lung volume variables should be monitored where possible (Fig. 2A).

Consensus was reached that a decline in DLco of 10–15% or a decline in FVC of 10% from baseline are indicators of progression of ILD, and that progression can be identified with lesser declines if trends are consistent across many variables (Fig. 2B). Consensus was also reached that acute exacerbations and worsening of dyspnoea are indicators of progression (Fig. 2B). There was consensus that otherwise unexplained worsening of cough or respiratory health status should prompt an evaluation for ILD progression (Fig. 2B).

Decline in FVC, decline in DLco and increased fibrosis on HRCT were ranked as the most important endpoints for determining progression of ILD (Fig. 2C; Supplementary Table 4). Consensus was reached that progression on HRCT or a decline in FVC ≥ 10% from baseline is sufficient to determine progression (Fig. 2C). There was consensus that deterioration in 6MWT distance can define progression in patients with IPF, but not other ILDs (Fig. S2 in Additional file 1). Consensus was reached that small declines in multiple endpoints (over an agreed timeframe) or a trend indicates progression, but that an isolated decline at a single point is not sufficient (Fig. 2C). There was consensus that, if CT is used to identify clinically significant progression, a case-by-case judgement should be made on whether the change is convincing (Fig. 2C). There was consensus that, when there is evidence of progression of ILD, alternative explanations that should be considered are heart failure/heart disease, pulmonary hypertension, infection, pulmonary embolism/venous thromboembolism, acute exacerbation, and systemic disease (Fig. 2C).

### Time periods for monitoring and defining progression of ILD

Regarding frequency of monitoring, consensus was reached that for the first two years, physicians should follow up with patients with ILD every 3–6 months (Fig. 3). There was consensus that a patient should be followed up more frequently if at higher risk of progression (Fig. 3). Consensus was reached that, where possible, DLco should be measured every 6 ± 3 months and 6MWT should be performed as needed but not at every visit (Fig. 3).

Regarding time periods that define progression, there was consensus that progression of ILD is progression irrespective of the time since diagnosis (Fig. 3). Consensus was not reached on whether there is a minimum or maximum period for assessing progression of ILD (Table S4 in Additional file 1). No consensus was reached on specific periods over which degrees of change in DLco, FVC, 6MWT distance, exercise capacity, quality of life, total lung volume, fibrosis on HRCT, oxygen use, weight, or symptoms can define progression (Table S5 in Additional file 1).

### Management of ILD

There was consensus that appropriate management of ILD depends on the underlying disease and that, for most ILDs, includes assessing for comorbidities, pneumococcal and influenza vaccination and pulmonary rehabilitation (Fig. 4). There was consensus that immunomodulatory therapy is appropriate for patients with interstitial pneumonia with autoimmune features (IPAF), mixed connective tissue disease-associated ILD and myositis-associated ILD (Fig. 4), but the threshold for consensus was not reached for RA-ILD (64.4%) or SSc-ILD (65.4%). Consensus was reached that the triggers for a change in management of patients with ILD are progression despite treatment, worsening FVC and/or DLco, starting or increasing supplemental oxygen, and development of pulmonary hypertension or exertional hypoxaemia (Fig. 4 and Table S6 in Additional file 1).

### Defining PPF

Consensus was reached that, considering PPF as defined in the international guideline or the phenomenon of progressive fibrosing ILD, ‘despite adequate management’ or ‘despite usual management’ should be included in the definition of progression (Fig. 5 and Table S7 in Additional file 1). There was consensus that general respirologists, specialists in related areas and policy makers/payers would find the guideline definition useful (Fig. 5), but consensus was not reached that this definition would be useful to ILD experts, healthcare professionals outside speciality fields, or patients (Table S7 in Additional file 1). Consensus was not reached on whether progression occurring within 12 months or over a longer interval should both be considered as PPF (Table S7 in Additional file 1).

## Discussion

We sought consensus among practising respiratory physicians on key questions relating to the monitoring and management of fibrosing ILD, and the prediction and identification of progression, using a modified Delphi approach.

The factors identified as risk factors for progression of ILD were largely consistent across ILDs and included the extent of disease on CT. While a UIP pattern on CT or histology was viewed as a risk factor for progression, consensus was reached that a biopsy to look for a UIP pattern should not be performed solely for prognostic reasons. Given the potential morbidity and mortality associated with surgical biopsy, and the paucity of data to suggest that lung biopsy improves outcomes [15–18], debate continues about the risk:benefit of surgical lung biopsy and cryobiopsy to inform differential diagnosis, management and prognosis of patients with ILDs. Consensus was not reached on whether bronchoalveolar lavage should be performed for prognostic reasons, with the respondents almost evenly split on this question. This uncertainty may reflect the conflicting findings in the literature, with divergent findings in patients with different types of ILD and across studies [19–21], and differences in expertise in how to interpret the findings of bronchoalveolar lavage.

Decline in lung function (FVC and/or DLco) and an increase in fibrosis on HRCT were ranked as the most important endpoints for determining progression of ILD. There was consensus that thresholds of decline in FVC of 10% or decline in DLco of 10–15% (without specification of whether these thresholds related to absolute or relative declines) are indicators of progression, and that progression can be identified with lesser declines if trends are observed across many variables. A decline in FVC % predicted of 10% has been included as a criterion for progression of ILD in several clinical guidelines and consensus statements [22–25], but there is evidence that a smaller decline in FVC is also associated with early mortality [26–28]. A decline in DLco of 10% or 15% has most commonly been used as the threshold to indicate progression of ILD [1, 25] but most guidelines include a decline in DLco as an indicator of ILD progression only in conjunction with a decline in FVC [22–24], likely reflecting the technical challenges associated with measuring DLco and that a decline in DLco may have other causes, such as pulmonary hypertension.

Consensus was reached that worsening of dyspnoea is an indicator of ILD progression and that unexplained worsening of cough is a reason to evaluate for ILD progression. Worsening of respiratory symptoms in patients with ILDs has been associated with an increased risk of mortality [29, 30], but on its own, may not be sufficient to define ILD progression, given the many reasons for development/worsening of respiratory symptoms and the challenges in measuring them. Consensus was reached that acute exacerbations are indicators of progression of ILD, but are not sufficient to define progression. Similarly, the international guideline did not include acute exacerbation in the definition of PPF, but recommended that patients be reassessed after an acute exacerbation to determine whether progression has occurred [1].

The respondents in our survey reached consensus that an increase in the extent of fibrosis on HRCT is sufficient to determine progression. This is in contrast to the international guideline criteria for PPF [1], and to the inclusion criteria used in clinical trials in PPF [2–5], which required that an individual fulfilled another criterion in addition to, or instead of, radiologic progression. Other than an increase in the extent of fibrosis, the changes on HRCT that would constitute progression on HRCT were not investigated in this study, but there was consensus that a case-by-case judgement should be made on whether changes on HRCT are convincing. It is challenging even for experienced radiologists to assess progression on HRCT based on visual assessment and changes in visually assessed CT scores may not correlate closely with changes in PFTs [31, 32]. Quantitative HRCT scores hold promise as reliable indicators of progression [33], but have not been studied extensively in patients with ILDs other than IPF and systemic sclerosis-associated ILD, and are not generally available in clinical practice.

Consensus was reached that a deterioration in 6MWT distance can define progression in patients with IPF, but not in patients with other ILDs. This reflects the literature: several studies have demonstrated that a decline in 6MWT distance is predictive of mortality in patients with IPF [34, 35], but there is a paucity of evidence to support this in patients with other ILDs.

In our study, there was consensus that for the first two years, patients with ILD should be followed up every 3–6 months, with more frequent monitoring within this time interval in patients at higher risk of progression. While there is a lack of evidence to link specific frequencies of follow-up to patient outcomes, a follow-up period of 3–6 months for patients with early ILD has been suggested by other experts in the field [9, 12, 25, 36]. Importantly, no consensus was reached on the time frame over which progression should occur to consider a patient as having progression. While time frames have been defined to identify patients with PPF for enrolment into clinical trials, these may not be appropriate for use in clinical practice. The argument has been made that progression is progression irrespective of the period over which it occurs [37]. This was reflected in the ATS guideline for management of SSc-ILD [38] and in the American College of Rheumatology guideline for the treatment of systemic autoimmune rheumatic disease-related ILDs [39], but not in the international guideline for definition of PPF, which specified that progression be assessed within a one-year period [1]. Progression occurring over a longer period may be clinically relevant, particularly in patients with a poor respiratory reserve. This applies especially to patients with combined pulmonary fibrosis and emphysema, in whom FVC decline is known to be attenuated, with attendant delays in identifying disease progression. Further, real-world delays in obtaining follow-up tests and variability in FVC measurements means that patients who are progressing may not meet criteria for progression if a strict time period is applied. It should also be borne in mind that progression of lung fibrosis is irreversible; thus “slow” progression over a prolonged period of time may ultimately become clinically significant.

The international guideline criteria for PPF did not include a requirement for a patient to have shown progression of ILD despite management, but provided a conditional recommendation for use of nintedanib, which has been licensed for the treatment of progressive fibrosing ILDs, in patients who have failed “standard management” for that ILD. In our study, consistent with other statements issued by expert groups [14, 23, 40], consensus was reached that ‘despite adequate management’ or ‘despite usual management’ should be included in the definition of ILD progression, but no information was collected on what the respondents believed would represent “adequate” or “usual” care. In practice, the standard of care for ILDs varies across types of ILD and according to patients’ individual needs and preferences. Importantly, for some patients, management may comprise monitoring without treatment; this applies to pneumoconioses such as asbestosis, pleuroparenchymal fibroelastosis (PPFE), or unclassifiable ILD and to some patients for whom the benefit of treatment is not deemed to outweigh the risks, based on joint decision-making with the patient.

Strengths of our study include the participation of a large number of physicians with varying clinical backgrounds. Limitations include that there are no standard criteria for defining consensus in Delphi studies, that most of the respondents were pulmonologists working at academic centres or teaching hospitals, and that a substantial proportion of physicians did not complete all three rounds of the survey. It is possible that the release of the international guidelines on the definition of PPF after the first round might have influenced responses in the second and third rounds. We acknowledge also that the participants consisted solely of clinicians and did not include radiologists and histopathologists participating in ILD multidisciplinary evaluation**.** The question of whether absolute or relative declines in FVC or DL_CO_ should be used to define progression of ILD was not investigated in our study.

## Conclusions

This modified Delphi process provided consensus statements on the identification of ILD progression that were supported by a broad group of clinicians and may help to inform clinical practice until more robust evidence-based guidelines are available.

## Supplementary Information

Supplementary Material 1.

## Data Availability

All data generated or analysed during this study are included in this published article and its supplementary information files.

## References

[1] Raghu G, Remy-Jardin M, Richeldi L, et al. Idiopathic pulmonary fibrosis (an update) and progressive pulmonary fibrosis in adults: an official ATS/ERS/JRS/ALAT clinical practice guideline. Am J Respir Crit Care Med. 2022;205:e18–47. 35486072 10.1164/rccm.202202-0399ST PMC9851481

[2] Flaherty KR, Wells AU, Cottin V, et al. Nintedanib in progressive fibrosing interstitial lung diseases. N Engl J Med. 2019;381:1718–27. 31566307 10.1056/NEJMoa1908681

[3] Maher TM, Corte TJ, Fischer A, et al. Pirfenidone in patients with unclassifiable progressive fibrosing interstitial lung disease: a double-blind, randomised, placebo-controlled, phase 2 trial. Lancet Respir Med. 2020;8:147–57. 31578169 10.1016/S2213-2600(19)30341-8

[4] Maher TM, Assassi S, Azuma A, et al. Design of a phase III, double-blind, randomised, placebo-controlled trial of BI 1015550 in patients with progressive pulmonary fibrosis (FIBRONEER-ILD). BMJ Open Respir Res. 2023;10: e001580. 37709661 10.1136/bmjresp-2022-001580 PMC10503394

[5] Behr J, Prasse A, Kreuter M, et al. Pirfenidone in patients with progressive fibrotic interstitial lung diseases other than idiopathic pulmonary fibrosis (RELIEF): a double-blind, randomised, placebo-controlled, phase 2b trial. Lancet Respir Med. 2021;9:476–86. 33798455 10.1016/S2213-2600(20)30554-3

[6] Brown KK, Inoue Y, Flaherty KR, et al. Predictors of mortality in subjects with progressive fibrosing interstitial lung diseases. Respirology. 2022;27:294–300. 35224814 10.1111/resp.14231 PMC9306931

[7] Hambly N, Farooqi MM, Dvorkin-Gheva A, et al. Prevalence and characteristics of progressive fibrosing interstitial lung disease in a prospective registry. Eur Respir J. 2022;60:2102571. 35273032 10.1183/13993003.02571-2021

[8] Oldham JM, Lee CT, Wu Z, et al. Lung function trajectory in progressive fibrosing interstitial lung disease. Eur Respir J. 2022;59:2101396. 34737223 10.1183/13993003.01396-2021 PMC10039317

[9] Hoffmann-Vold A-M, Maher TM, Philpot EE, et al. The identification and management of interstitial lung disease in systemic sclerosis: evidence-based European consensus statements. Lancet Rheumatol. 2020;2:e71–83. 38263663 10.1016/S2665-9913(19)30144-4

[10] Fernández Pérez ER, Koelsch TL, Leone PM, Groshong SD, Lynch DA, Brown KK. Clinical decision-making in hypersensitivity pneumonitis: diagnosis and management. Semin Respir Crit Care Med. 2020;41:214–28. 32279292 10.1055/s-0040-1701250

[11] Bendstrup E, Kronborg-White S, Møller J, Prior TS. Current best clinical practices for monitoring of interstitial lung disease. Expert Rev Respir Med. 2022;16:1153–66. 36572644 10.1080/17476348.2022.2162504

[12] Case AH, Beegle S, Hotchkin DL, et al. Defining the pathway to timely diagnosis and treatment of interstitial lung disease: a US Delphi survey. BMJ Open Respir Res. 2023;10: e001594. 38007235 10.1136/bmjresp-2022-001594 PMC10680004

[13] Rahaghi FF, Hsu VM, Kaner RJ, et al. Expert consensus on the management of systemic sclerosis-associated interstitial lung disease. Respir Res. 2023;24:6. 36624431 10.1186/s12931-022-02292-3 PMC9830797

[14] Rajan SK, Cottin V, Dhar R, et al. Progressive pulmonary fibrosis: an expert group consensus statement. Eur Respir J. 2023;61:2103187. 36517177 10.1183/13993003.03187-2021 PMC10060665

[15] Hariri LP, Roden AC, Chung JH, Danoff SK, et al. The role of surgical lung biopsy in the diagnosis of fibrotic interstitial lung disease: perspective from the Pulmonary Fibrosis Foundation. Ann Am Thorac Soc. 2021;18:1601–9. 34004127 10.1513/AnnalsATS.202009-1179FR

[16] Ravaglia C, Nicholson AG. Biopsy in interstitial lung disease: specific diagnosis and the identification of the progressive fibrotic phenotype. Curr Opin Pulm Med. 2021;27:355–62. 34397611 10.1097/MCP.0000000000000810

[17] Korevaar DA, Colella S, Fally M, et al. European Respiratory Society guidelines on transbronchial lung cryobiopsy in the diagnosis of interstitial lung diseases. Eur Respir J. 2022;60:2200425. 35710261 10.1183/13993003.00425-2022

[18] Damiani A, Orlandi M, Bruni C, et al. The role of lung biopsy for diagnosis and prognosis of interstitial lung disease in systemic sclerosis: a systematic literature review. Respir Res. 2024;25:138. 38521926 10.1186/s12931-024-02725-1 PMC10960984

[19] Bowman WS, Echt GA, Oldham JM. Biomarkers in progressive fibrosing interstitial lung disease: optimizing diagnosis, prognosis, and treatment response. Front Med (Lausanne). 2021;8: 680997. 34041256 10.3389/fmed.2021.680997 PMC8141562

[20] Tomassetti S, Colby TV, Wells AU, Poletti V, Costabel U, Matucci-Cerinic M. Bronchoalveolar lavage and lung biopsy in connective tissue diseases, to do or not to do? Ther Adv Musculoskelet Dis. 2021;13:1759720X211059605. 34900002 10.1177/1759720X211059605 PMC8664307

[21] Barnett JL, Maher TM, Quint JK, et al. Combination of BAL and computed tomography differentiates progressive and non-progressive fibrotic lung diseases. Am J Respir Crit Care Med. 2023;208:975–82. 37672028 10.1164/rccm.202305-0796OC

[22] Khanna D, Mittoo S, Aggarwal R, et al. Connective tissue disease-associated interstitial lung diseases (CTD-ILD)—report from OMERACT CTD-ILD working group. J Rheumatol. 2015;42:2168–71. 25729034 10.3899/jrheum.141182 PMC4809413

[23] George PM, Spagnolo P, Kreuter M, et al. Progressive fibrosing interstitial lung disease: clinical uncertainties, consensus recommendations, and research priorities. Lancet Respir Med. 2020;8:925–34. 32890499 10.1016/S2213-2600(20)30355-6

[24] Piotrowski WJ, Martusewicz-Boros MM, Białas AJ, et al. Guidelines of the Polish Respiratory Society on the diagnosis and treatment of progressive fibrosing interstitial lung diseases other than idiopathic pulmonary fibrosis. Adv Respir Med. 2022;90:425–50. 36285980 10.3390/arm90050052 PMC9717335

[25] Radić M, Novak S, Barešić M, et al. Delphi-based consensus on interstitial lung disease screening in patients with connective tissue diseases (Croatian national-based study). Biomedicines. 2022;10:3291. 36552047 10.3390/biomedicines10123291 PMC9775485

[26] Zappala CJ, Latsi PI, Nicholson AG, et al. Marginal decline in forced vital capacity is associated with a poor outcome in idiopathic pulmonary fibrosis. Eur Respir J. 2010;35:830–6. 19840957 10.1183/09031936.00155108

[27] du Bois RM, Weycker D, Albera C, et al. Forced vital capacity in patients with idiopathic pulmonary fibrosis: test properties and minimal clinically important difference. Am J Respir Crit Care Med. 2011;184:1382–9. 21940789 10.1164/rccm.201105-0840OC

[28] Maher TM, Stowasser S, Voss F, et al. Decline in forced vital capacity as a surrogate for mortality in patients with pulmonary fibrosis. Respirology. 2023;28:1147–53. 37646126 10.1111/resp.14579

[29] Case AH, Hellkamp AS, Neely ML, et al. Associations between patient-reported outcomes and death or lung transplant in idiopathic pulmonary fibrosis. Data from the Idiopathic Pulmonary Fibrosis Prospective Outcomes Registry. Ann Am Thorac Soc. 2020;17:699–705. 32040340 10.1513/AnnalsATS.201906-437OC PMC7258421

[30] Lee J, White E, Freiheit E, et al. Cough-specific quality of life predicts disease progression among patients with interstitial lung disease: data from the Pulmonary Fibrosis Foundation Patient Registry. Chest. 2022;162:603–13. 35337809 10.1016/j.chest.2022.03.025 PMC9808640

[31] Taha N, D’Amato D, Hosein K, Ranalli T, Sergiacomi G, Zompatori M, Mura M. Longitudinal functional changes with clinically significant radiographic progression in idiopathic pulmonary fibrosis: are we following the right parameters? Respir Res. 2020;21:119. 32429952 10.1186/s12931-020-01371-7 PMC7238541

[32] Carnevale A, Silva M, Maietti E, et al. Longitudinal change during follow-up of systemic sclerosis: correlation between high-resolution computed tomography and pulmonary function tests. Clin Rheumatol. 2021;40:213–9. 32880053 10.1007/s10067-020-05375-y

[33] Walsh SLF, De Backer J, Prosch H, et al. Towards the adoption of quantitative computed tomography in the management of interstitial lung disease. Eur Respir Rev. 2024;33: 230055. 38537949 10.1183/16000617.0055-2023 PMC10966471

[34] du Bois RM, Weycker D, Albera C, et al. Six-minute-walk test in idiopathic pulmonary fibrosis: test validation and minimal clinically important difference. Am J Respir Crit Care Med. 2011;183:1231–7. 21131468 10.1164/rccm.201007-1179OC

[35] Nathan SD, du Bois RM, Albera C, et al. Validation of test performance characteristics and minimal clinically important difference of the 6-minute walk test in patients with idiopathic pulmonary fibrosis. Respir Med. 2015;109:914–22. 25956020 10.1016/j.rmed.2015.04.008

[36] Johnson SR, Bernstein EJ, Bolster MB, et al. 2023 American College of Rheumatology (ACR)/American College of Chest Physicians (CHEST) guideline for the screening and monitoring of interstitial lung disease in people with systemic autoimmune rheumatic diseases. Arthritis Care Res (Hoboken) 2024. 10.1002/acr.25347. 10.1002/acr.25347 PMC12646460 38973729

[37] Cottin V, Brown KK, Flaherty KR, Wells AU. Progressive pulmonary fibrosis: should the timelines be taken out of the definition? Am J Respir Crit Care Med. 2022;206:1293–4. 35868029 10.1164/rccm.202206-1143LE PMC9746830

[38] Raghu G, Montesi SB, Silver RM, et al. Treatment of systemic sclerosis-associated interstitial lung disease: evidence-based recommendations. An official American Thoracic Society clinical practice guideline. Am J Respir Crit Care Med. 2024;209:137–52. 37772985 10.1164/rccm.202306-1113ST PMC10806429

[39] Johnson SR, Bernstein EJ, Bolster MB, et al. American College of Rheumatology (ACR)/American College of Chest Physicians (CHEST) guideline for the treatment of interstitial lung disease in people with systemic autoimmune rheumatic diseases. Arthritis Care Res (Hoboken). 2023. 10.1002/acr.25348. 37394710 10.1002/acr.25184 PMC10758525

[40] Mackintosh JA, Keir G, Troy LK, et al. Treatment of idiopathic pulmonary fibrosis and progressive pulmonary fibrosis: a position statement from the Thoracic Society of Australia and New Zealand 2023 revision. Respirology. 2024;29:105–35. 38211978 10.1111/resp.14656 PMC10952210

