# Supplementary material for: Identification of progressive pulmonary fibrosis: consensus findings from a modified Delphi study
Source: Respir Res. 2024 Dec 31;25:448. doi: 10.1186/s12931-024-03070-z (PMC11687192; doi:10.1186/s12931-024-03070-z)
Supplement: Supplementary file 1 — Supplementary Material 1. [file 12931_2024_3070_MOESM1_ESM.docx]

**Additional file 1.**

**Fig. S1** Consensus statements on risk factors for progression of individual ILDs.


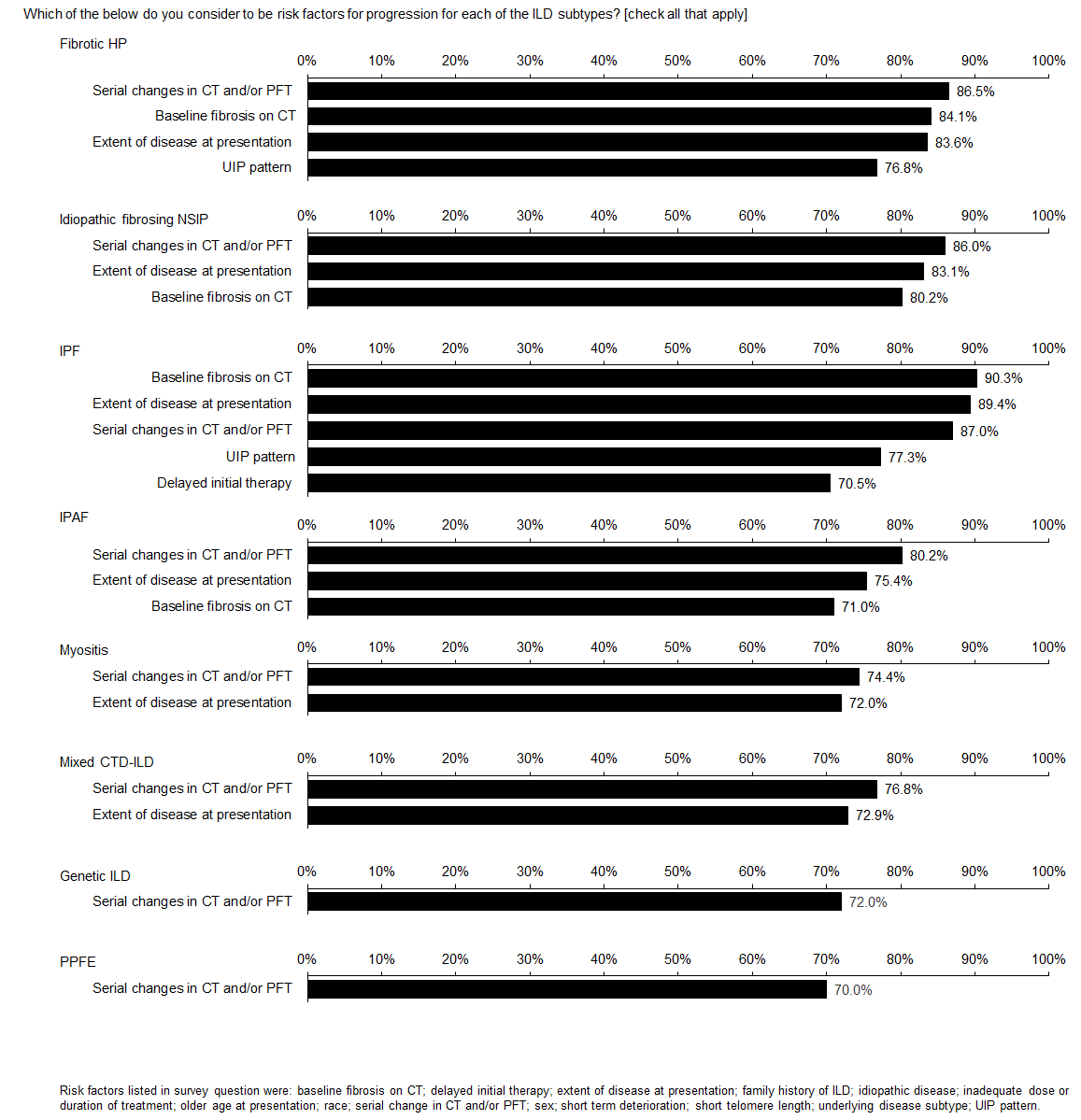


**Fig S2**  Consensus statements on factors that define progression of individual ILDs.


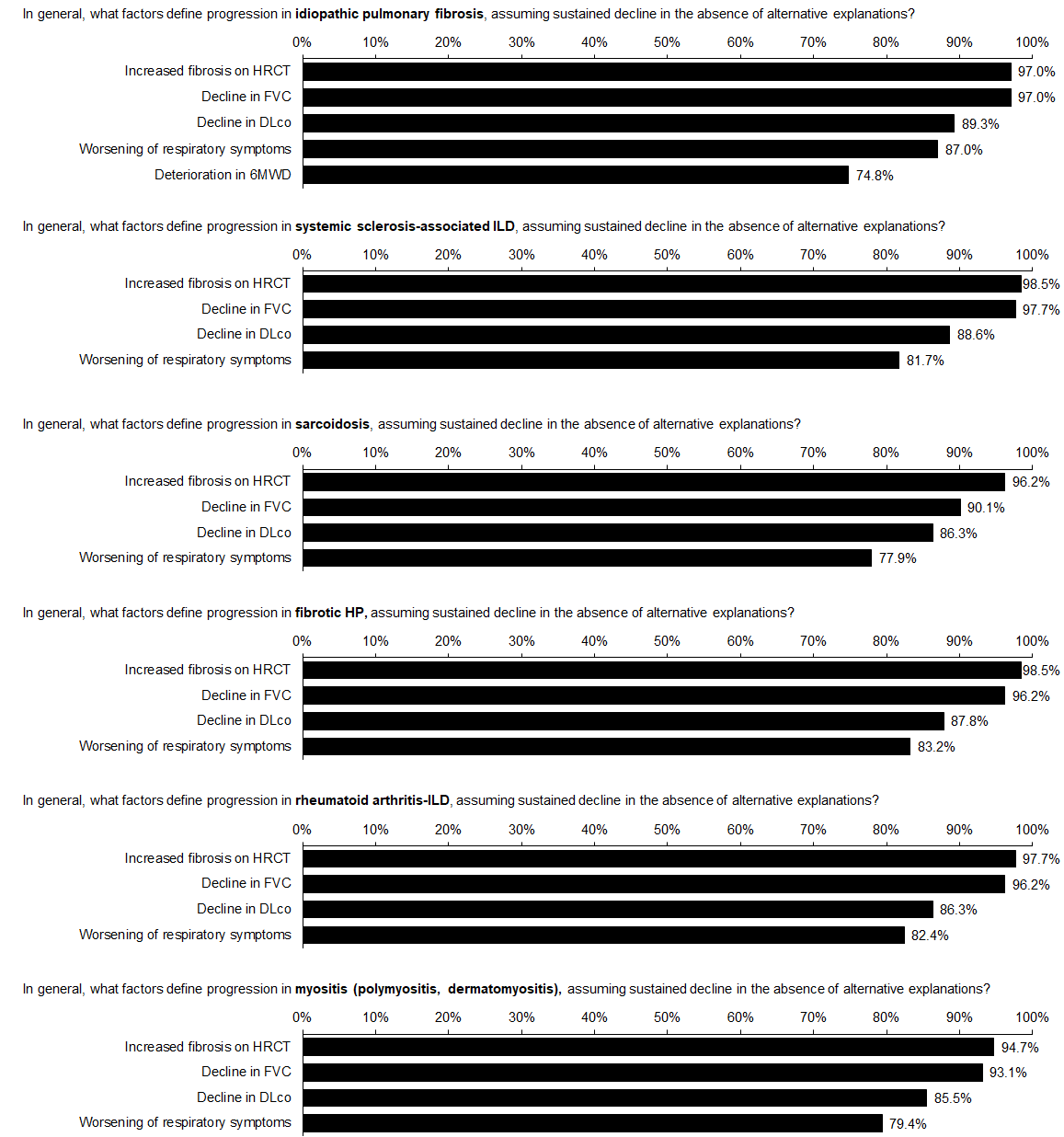


**Table S1** Clinical experience of physicians who participated in the surveys.

|  | **Survey 1**  **(N=207)** | **Survey 2**  **(N=131)** | **Survey 3**  **(N=94)** |
| --- | --- | --- | --- |
| **Clinical specialty** |  |  |  |
| Pulmonology | 202 (97.6) | 128 (97.7) | 93 (98.9) |
| Internal medicine | 3 (1.5) | 2 (1.5) | 1 (1.1) |
| Rheumatology | 1 (0.5) | 0 | 0 |
| Pulmonology and allergology | 1 (0.5) | 1 (0.8) | 0 |
| **Main practice** |  |  |  |
| Academic centre/teaching hospital | 178 (86.0) | 114 (87.0) | 83 (88.3) |
| General non-teaching hospital | 13 (6.3) | 6 (4.6) | 3 (3.2) |
| Private practice primary care | 11 (5.3) | 7 (5.3) | 5 (5.3) |
| Private practice, reference speciality | 2 (1.0) | 0 | 0 |
| Private practice and general hospital | 1 (0.5) | 0 | 0 |
| Chest clinic | 1 (0.5) | 0 | 0 |
| Outpatient pulmonary clinic | 1 (0.5) | 0 | 0 |
| Other | 0 | 4 (3.1%) | 3 (3.2) |
| **Years of experience in clinical speciality** |  |  |  |
| <5 | 7 (3.4) | 3 (2.3) | 94 (100.0) |
| >5–≤10 | 24 (11.6) | 15 (11.5) | 0 |
| >10–≤15 | 44 (21.3) | 27 (20.6) | 0 |
| >15–≤20 | 40 (19.3) | 27 (20.6) | 0 |
| >20–≤25 | 39 (18.8) | 25 (19.1) | 0 |
| >25 | 53 (25.6) | 34 (26.0) | 0 |
| **Patients with ILD diagnosed/treated in last 12 months** |  |  |  |
| 1–50 | 47 (22.7) | 25 (19.1) | 17 (18.1) |
| 51–100 | 38 (18.4) | 15 (11.5) | 18 (19.1) |
| 101–150 | 27 (13.0) | 24 (18.3) | 10 (10.6) |
| 151–200 | 16 (7.7) | 15 (11.5) | 13 (13.8) |
| >200 | 79 (38.2) | 52 (39.7) | 36 (38.3) |

Data are n (%).

**Table S2** Questions and responses on “predicting progression” in **A**) survey 1, **B**) survey 2 and **C**) survey 3.

**A**

| **Question/consensus statement** | **n** | **Response** | **% of respondents** | **Median and IQR for responses on Likert scale** | **Consensus?** |
| --- | --- | --- | --- | --- | --- |
| How important is it to define risk factors for progression of ILD?* | 207 | Extremely important | 58.5% | Median = 3  IQR = 1 | Yes |
|  |  | Important | 36.2% |  |  |
|  |  | Slightly important | 4.8% |  |  |
|  |  | Neutral | 0% |  |  |
|  |  | Slightly not important | 0% |  |  |
|  |  | Not important | 0.5% |  |  |
|  |  | Not at all important | 0% |  |  |
| What do you consider to be risk factors for progression of ILD? | 207 | Extent of disease on CT at presentation | 86.0% | n/a | Yes |
|  |  | UIP pattern on CT | 86.0% |  |  |
|  |  | Baseline fibrosis pattern on CT | 84.1% |  |  |
|  |  | Poor pulmonary function at presentation | 84.1% |  |  |
|  |  | Family history of ILD | 66.7% |  |  |
|  |  | Short-term deterioration | 63.3% |  |  |
|  |  | Short telomere length | 59.9% |  |  |
|  |  | Non-adherence to treatment | 59.4% |  |  |
|  |  | Delayed initial therapy | 55.1% |  |  |
|  |  | Idiopathic disease | 53.6% |  |  |
|  |  | Current or ex-smoker | 53.1% |  |  |
|  |  | Genetic markers/biomarkers | 49.3% |  |  |
|  |  | Autoantibody status | 45.4% |  |  |
|  |  | Older age at presentation | 44.4% |  |  |
| Which of the below do you consider to be **risk factors for progression for each of the ILD subtypes?^†^** [only responses that >70% participants checked are shown] | 207 | Baseline fibrosis on CT is a risk factor for progression for fibrotic HP | 84.1% | n/a | Yes |
|  |  | Baseline fibrosis on CT is a risk factor for progression for idiopathic fibrosing NSIP | 80.2% |  |  |
|  |  | Baseline fibrosis on CT is a risk factor for progression for IPF | 90.3% |  |  |
|  |  | Baseline fibrosis on CT is a risk factor for progression for IPAF | 71.0% |  |  |
|  |  | Delayed initial therapy is a risk factor for progression for IPF | 70.5% |  |  |
|  |  | Extent of disease at presentation is a risk factor for progression for fibrotic HP | 83.6% |  |  |
|  |  | Extent of disease at presentation is a risk factor for progression for idiopathic fibrosing NSIP | 83.1% |  |  |
|  |  | Extent of disease at presentation is a risk factor for progression for IPF | 89.4% |  |  |
|  |  | Extent of disease at presentation is a risk factor for progression for IPAF | 75.4% |  |  |
|  |  | Extent of disease at presentation is a risk factor for progression for mixed CTD-ILD | 72.9% |  |  |
|  |  | Extent of disease at presentation is a risk factor for progression for myositis | 72.0% |  |  |
|  |  | Serial changes in CT and/or PFT is a risk factor for progression for fibrotic HP | 86.5% |  |  |
|  |  | Serial changes in CT and/or PFT is a risk factor for progression for genetic ILD(e.g. Hermansky-Pudlak syndrome, short telomere, etc.) | 72.0% |  |  |
|  |  | Serial changes in CT and/or PFT is a risk factor for progression for idiopathic fibrosing NSIP | 86.0% |  |  |
|  |  | Serial changes in CT and/or PFT is a risk factor for progression for IPF | 87.0% |  |  |
|  |  | Serial changes in CT and/or PFT is a risk factor for progression for IPAF | 80.2% |  |  |
|  |  | Serial changes in CT and/or PFT is a risk factor for progression for mixed CTD-ILD | 76.8% |  |  |
|  |  | Serial changes in CT and/or PFT is a risk factor for progression for myositis (polymyositis, dermatomyositis) | 74.4% |  |  |
|  |  | Serial changes in CT and/or PFT is a risk factor for progression for pleuroparenchymal fibroelastosis (PPFE) | 70.0% |  |  |
|  |  | UIP pattern is a risk factor for progression for fibrotic HP | 76.8% |  |  |
|  |  | UIP pattern is a risk factor for progression for IPF | 77.3% |  |  |
| Which underlying diagnoses do you consider as having the highest risk for progression of ILD despite management?^†^ | 207 | IPF | 89.9% | n/a | Yes |
|  |  | Fibrotic HP | 61.8% |  |  |
|  |  | Genetic ILD (e.g. Hermansky-Pudlak syndrome, short telomere, etc.) | 54.1% |  |  |
|  |  | PPFE | 44.9% |  |  |
|  |  | RA-ILD | 43.5% |  |  |
|  |  | SSc-ILD | 41.6% |  |  |
|  |  | Unclassifiable ILD/IIP | 41.1% |  |  |
|  |  | Idiopathic fibrosing NSIP | 37.2% |  |  |
|  |  | Myositis (polymyositis, dermatomyositis) | 27.5% |  |  |
|  |  | Mixed CTD-ILD | 18.4% |  |  |
|  |  | Pneumoconiosis | 11.6% |  |  |
|  |  | Sjögren’s disease | 8.2% |  |  |
|  |  | Sarcoidosis | 5.3% |  |  |
|  |  | Post-infection ILD | 1.9% |  |  |
|  |  | Other (please specify) | 2.4% |  |  |
|  |  | Fibrotic HP with honeycombing | 0.5% |  |  |
|  |  | Any underlying disease with UIP pattern | 0.5% |  |  |
|  |  | CTD-UIP | 0.5% |  |  |
|  |  | Reason differs depending on diagnosis | 0.5% |  |  |
|  |  | Additional comment | 0.5% |  |  |
| When there is evidence of disease progression, what scenarios would you consider as alternative explanations of disease worsening, other than progression of pulmonary fibrosis?^†^ | 207 | Heart failure/heart disease | 90.8% | n/a | Yes |
|  |  | Pulmonary hypertension | 89.4% |  |  |
|  |  | Infection | 84.1% |  |  |
|  |  | Pulmonary embolism/venous thromboembolism | 82.1% |  |  |
|  |  | Acute exacerbation | 80.7% |  |  |
|  |  | Activity of underlying systemic disease | 73.0% |  |  |
|  |  | Drug toxicity | 69.6% |  |  |
|  |  | Aspiration | 66.2% |  |  |
|  |  | Progression of inflammatory lung disease | 63.8% |  |  |
|  |  | Cancer | 60.4% |  |  |
|  |  | Deconditioning | 58.0% |  |  |
|  |  | Other comorbidities | 44.0% |  |  |
|  |  | Unsure/not applicable | 0.5% |  |  |
|  |  | Other | 3.9% |  |  |
| Is the evidence supporting any blood-based biomarkers of progression in ILD sufficient to be used in clinical practice? | 207 | Yes | 21.3% | n/a | Yes |
|  |  | No | 78.7% |  |  |
| Do you collect/use any blood-based biomarkers in clinical practice? | 207 | Yes | 30.0% | n/a | Yes |
|  |  | No | 70.1% |  |  |
| Do you use biopsies performed for diagnostic purposes to also predict risk of progression? | 207 | Yes | 36.2% | n/a | No |
|  |  | No | 63.8% |  |  |
| If yes, in what situation would you use biopsies performed for diagnostic purposes to also predict risk of progression?^§^ | 73 | Any/all cases | 2.4% | n/a | No |
|  |  | IPF | 2.4% |  |  |
|  |  | IPAF | 1.4% |  |  |
|  |  | Fibroblastic foci | 1.9% |  |  |
|  |  | HP | 3.9% |  |  |
|  |  | NSIP | 1.9% |  |  |
|  |  | Unclassifiable | 4.8% |  |  |
|  |  | UIP | 6.8% |  |  |
|  |  | CTD | 1.9% |  |  |
|  |  | If unsure/support diagnosis | 9.7% |  |  |
|  |  | Support treatment decisions | 1.0% |  |  |
|  |  | Other | 2.4% |  |  |
| If yes, which histopathological patterns in ILD would you consider as a predictor for risk of progression?^§^ | 73 | UIP | 29.5% | n/a | No |
|  |  | Fibroblast | 8.2% |  |  |
|  |  | HP | 3.4% |  |  |
|  |  | NSIP | 2.9% |  |  |
|  |  | Sarcoidosis | 1.0% |  |  |
| Do you use bronchoalveolar lavage performed for diagnostic purposes to also predict risk of progression? | 207 | Yes | 40.6% | n/a | No |
|  |  | No | 59.4% |  |  |
| If yes, in what situation would you perform bronchoalveolar lavage?^§^ | 83 | All cases | 4.3% | n/a | No |
|  |  | Aid diagnosis | 23.7% |  |  |
|  |  | Inflammatory disease | 2.4% |  |  |
|  |  | Clinical decline | 2.9% |  |  |
|  |  | Biopsy not possible | 1.4% |  |  |
|  |  | Exclude infection | 5.8% |  |  |
|  |  | Treatment decision | 1.0% |  |  |
|  |  | Other | 1.9% |  |  |
| If yes, which histopathological patterns in ILD would you consider as a predictor for risk of progression?^§^ | 78 | UIP | 15.9% | n/a | No |
|  |  | NSIP | 1.0% |  |  |
|  |  | High number of fibroblastic foci | 1.9% |  |  |
|  |  | Neutrophilic BAL | 9.2% |  |  |
|  |  | No lymphocytosis | 5.8% |  |  |
|  |  | Other | 7.2% |  |  |

*Statements were assessed on a 7-point Likert scale. The scale was from -3 (not at all important/strongly disagree) to +3 (extremely important/strongly agree). Data are % of respondents that selected each Likert scale category.

^†^More than one answer could be selected.

^‡^Data shown are median ranking/score.

^§^Free text responses.

CT, computed tomography; CTD-ILD, connective tissue disease-associated interstitial lung disease; HP, hypersensitivity pneumonitis IIP, idiopathic interstitial pneumonia; ILD, interstitial lung disease; IPAF, interstitial pneumonia with autoimmune features; IPF, idiopathic pulmonary fibrosis; NSIP, non-specific interstitial pneumonia; PFT, pulmonary function test; PPFE, pleuroparenchymal fibroelastosis; RA-ILD, rheumatoid arthritis-associated interstitial lung disease; SSc-ILD scleroderma-associated interstitial lung disease; UIP, usual interstitial pneumonia.

**B**

| **Question/consensus statement** | **n** | **Response** | **% of respondents** | **Median and IQR for responses on Likert scale** | **Consensus?** |
| --- | --- | --- | --- | --- | --- |
| Please rank the following disorders by risk of progression^‡^ | 131 | Ranking from most to least important:^‡^ |  |  |  |
|  |  | Genetic ILD (e.g. Hermansky–Pudlak syndrome, short telomere, etc.)/familial pulmonary fibrosis |  | 3.0 | n/a |
|  |  | Fibrotic HP |  | 3.0 |  |
|  |  | Idiopathic fibrosing NSIP |  | 5.0 |  |
|  |  | Unclassifiable ILD/IIPs |  | 5.0 |  |
|  |  | SSc-ILD |  | 5.0 |  |
|  |  | RA-ILD |  | 5.0 |  |
|  |  | Pleuroparenchymal fibroelastosis |  | 6.0 |  |
|  |  | Undifferentiated CTD-ILD/ IPAF |  | 7.0 |  |
|  |  | Myositis (polymyositis, dermatomyositis) |  | 7.0 |  |
|  |  | Sjögren’s disease |  | 9.0 |  |
|  |  | Sarcoidosis |  | 11.0 |  |
|  |  | Pneumoconiosis |  | 11.0 |  |
|  |  | Post-infection ILD |  | 13.0 |  |
| Would you perform a biopsy solely for prognostic reasons in a patient with the presence of honeycombing on CT? | 131 | Yes | 7.6% | n/a | Yes |
|  |  | No | 92.4% |  |  |
| If yes, when performing a biopsy solely for prognostic reasons in a patient with the presence of honeycombing on CT, would you use surgical lung biopsy? | 8 | Yes | 62.5% | n/a | No |
|  |  | No | 37.5% |  |  |
| If yes, when performing a biopsy solely for prognostic reasons in a patient with the presence of honeycombing on CT, would you use cryobiopsy? | 9 | Yes | 100% | n/a | No |
|  |  | No | 0% |  |  |
| If yes, when performing a biopsy solely for prognostic reasons in a patient with the presence of honeycombing on CT, would you use transbronchial biopsy with the genomic classifier? | 9 | Yes | 44.4% | n/a | No |
|  |  | No | 55.6% |  |  |
| Would you perform a biopsy solely for prognostic reasons in a patient with a pattern on CT that is indeterminate for UIP in order to look for a UIP pattern? | 131 | Yes | 14.5% | n/a | Yes |
|  |  | No | 85.5% |  |  |
| If yes, when performing a biopsy solely for prognostic reasons in a patient with a pattern on CT that is indeterminate for UIP, would you use surgical lung biopsy in order to look for a UIP pattern? | 15 | Yes | 93.3% | n/a | No |
|  |  | No | 6.7% |  |  |
| If yes, when performing a biopsy solely for prognostic reasons in a patient with a pattern on CT that is indeterminate for UIP, would you use cryobiopsy in order to look for a UIP pattern? | 17 | Yes | 82.4% | n/a | No |
|  |  | No | 17.7% |  |  |
| If yes, when performing a biopsy solely for prognostic reasons in a patient with a pattern on CT that is indeterminate for UIP, would you use transbronchial biopsy with the genomic classifier in order to look for a UIP pattern? | 14 | Yes | 28.6% | n/a | No |
|  |  | No | 71.4% |  |  |
| Would you perform a biopsy solely for prognostic reasons in a patient with CT appearances that are clearly atypical for UIP in order to look for a UIP pattern? | 131 | Yes | 61.8% | n/a | No |
|  |  | No | 38.2% |  |  |
| If yes, when performing a biopsy solely for prognostic reasons in a patient with CT appearances that are clearly atypical for UIP, would you use surgical lung biopsy in order to look for a UIP pattern? | 77 | Yes | 81.8% | n/a | No |
|  |  | No | 18.2% |  |  |
| If yes, when performing a biopsy solely for prognostic reasons in a patient with CT appearances that are clearly atypical for UIP, would you use cryobiopsy in order to look for a UIP pattern? | 75 | Yes | 78.7% | n/a | No |
|  |  | No | 21.3% |  |  |
| If yes, when performing a biopsy solely for prognostic reasons in a patient with CT appearances that are clearly atypical for UIP, would you use transbronchial biopsy with the genomic classifier in order to look for a UIP pattern? | 68 | Yes | 20.6% | n/a | No |
|  |  | No | 79.4% |  |  |
| Would you perform a biopsy solely for prognostic reasons in a patient with CT appearances that are clearly atypical for UIP in order to look for a UIP pattern? | 131 | Yes | 58.8% | n/a | No |
|  |  | No | 41.2% |  |  |
| If yes, when performing a biopsy solely for prognostic reasons in a patient with CT appearances that are clearly atypical for UIP, would you use surgical lung biopsy in order to look for a UIP pattern? | 71 | Yes | 80.3% | n/a | No |
|  |  | No | 19.7% |  |  |
| If yes, when performing a biopsy solely for prognostic reasons in a patient with CT appearances that are clearly atypical for UIP, would you use cryobiopsy in order to look for a UIP pattern? | 70 | Yes | 80.0% | n/a | No |
|  |  | No | 20.0% |  |  |
| If yes, when performing a biopsy solely for prognostic reasons in a patient with CT appearances that are clearly atypical for UIP, would you use transbronchial biopsy with the genomic classifier in order to look for a UIP pattern? | 60 | Yes | 23.0% | n/a | No |
|  |  | No | 77.1% |  |  |
| A bronchoalveolar lavage should be performed for prognostic reasons* | 131 | Strongly agree | 6.1% | Median = 0  IQR = 3 | No |
|  |  | Agree | 19.9% |  |  |
|  |  | Somewhat agree | 22.9% |  |  |
|  |  | Neutral | 13.7% |  |  |
|  |  | Somewhat disagree | 15.3% |  |  |
|  |  | Disagree | 13.7% |  |  |
|  |  | Strongly disagree | 8.4% |  |  |
| A UIP histological pattern should be considered as a predictor for risk of progression* | 131 | Strongly agree | 47.3% | Median = 2  IQR = 1 | Yes |
|  |  | Agree | 42.8% |  |  |
|  |  | Somewhat agree | 8.4% |  |  |
|  |  | Neutral | 0.8% |  |  |
|  |  | Somewhat disagree | 0.8% |  |  |
|  |  | Disagree | 0% |  |  |
|  |  | Strongly disagree | 0% |  |  |
|  |  | Agree | 48.9% |  |  |
|  |  | Somewhat agree | 17.0% |  |  |
|  |  | Neutral | 2.1% |  |  |
|  |  | Somewhat disagree | 3.2% |  |  |
|  |  | Disagree | 0% |  |  |
|  |  | Strongly disagree | 0% |  |  |

*Statements were assessed on a 7-point Likert scale. The scale was from -3 (not at all important/strongly disagree) to +3 (extremely important/strongly agree). Data are % of respondents that selected each Likert scale category.

^†^More than one answer could be selected.

^‡^Data shown are median ranking/score.

^§^Free text responses.

CT, computed tomography; CTD-ILD, connective tissue disease-associated interstitial lung disease; HP, hypersensitivity pneumonitis IIP, idiopathic interstitial pneumonia; ILD, interstitial lung disease; IPAF, interstitial pneumonia with autoimmune features; IPF, idiopathic pulmonary fibrosis; NSIP, non-specific interstitial pneumonia; PFT, pulmonary function test; PPFE, pleuroparenchymal fibroelastosis; RA-ILD, rheumatoid arthritis-associated interstitial lung disease; SSc-ILD scleroderma-associated interstitial lung disease; UIP, usual interstitial pneumonia.

**C**

| **Question/consensus statement** | **n** | **Response** | **% of respondents** | **Median and IQR for responses on Likert scale** | **Consensus?** |
| --- | --- | --- | --- | --- | --- |
| ***Survey 3*** | | | | |  |
| To what extent do you agree with the following: “In general, the disorders with highest risk of progression are: fibrotic HP, genetic ILD (e.g. short telomere, etc.)/familial pulmonary fibrosis, RA-ILD, SSc-ILD, unclassifiable ILD/IIPs and idiopathic fibrosing NSIP”?* | 94 | Strongly agree | 28.7% | Median = 2  IQR = 1 | Yes |
|  |  | Agree | 48.9% |  |  |
|  |  | Somewhat agree | 17.0% |  |  |
|  |  | Neutral | 2.1% |  |  |
|  |  | Somewhat disagree | 3.2% |  |  |
|  |  | Disagree | 0% |  |  |
|  |  | Strongly disagree | 0% |  |  |

*Statements were assessed on a 7-point Likert scale. The scale was from -3 (not at all important/strongly disagree) to +3 (extremely important/strongly agree). Data are % of respondents that selected each Likert scale category.

^†^More than one answer could be selected.

^‡^Data shown are median ranking/score.

^§^Free text responses.

CT, computed tomography; CTD-ILD, connective tissue disease-associated interstitial lung disease; HP, hypersensitivity pneumonitis IIP, idiopathic interstitial pneumonia; ILD, interstitial lung disease; IPAF, interstitial pneumonia with autoimmune features; IPF, idiopathic pulmonary fibrosis; NSIP, non-specific interstitial pneumonia; PFT, pulmonary function test; PPFE, pleuroparenchymal fibroelastosis; RA-ILD, rheumatoid arthritis-associated interstitial lung disease; SSc-ILD scleroderma-associated interstitial lung disease; UIP, usual interstitial pneumonia.

**Table S3** Questions and responses on “monitoring for progression” in **A**) survey 1, **B**) survey 2 and **C**) survey 3.

**A**

| **Question/consensus statement** | **n** | **Response** | **% of respondents** | **Median and IQR for responses on Likert scale** | **Consensus?** |
| --- | --- | --- | --- | --- | --- |
| In general, how often do you follow up with your patients with ILD within the first 2 years of management? | 207 | Every 3-4 months | 66.2% | n/a | Yes |
|  |  | Every 6 months | 19.3% |  |  |
|  |  | Every year | 0% |  |  |
|  |  | As indicated by changes in symptoms | 1.9% |  |  |
|  |  | It depends on what risk factors they have [please specify] | 4.4% |  |  |
|  |  | It depends on the specific disease (please specify) | 5.3% |  |  |
|  |  | Other (please specify) | 2.9% |  |  |
| Is there a maximum period of time you would monitor a patient with ILD for before deciding that the disease is not progressing? | 207 | 12 months | 23.7% | n/a | No |
|  |  | 18 months | 1.5% |  |  |
|  |  | 24 months | 17.9% |  |  |
|  |  | 36 months | 9.2% |  |  |
|  |  | 48 months | 4.8% |  |  |
|  |  | No maximum | 36.7% |  |  |
|  |  | Other (please specify) | 6.3% |  |  |
| In normal clinical practice, how important is **acute exacerbation** in monitoring for progression in ILD, based on sensitivity and accuracy?* | 207 | Very important | 59.9% | Median = 3  IQR = 1 | Yes |
|  |  | Important | 25.1% |  |  |
|  |  | Somewhat important | 9.2% |  |  |
|  |  | Neutral | 4.4% |  |  |
|  |  | Slightly not important | 1.0% |  |  |
|  |  | Not important | 0.5% |  |  |
|  |  | Not important at all | 0% |  |  |
| In normal clinical practice, how important is **decline in DLco** in monitoring for progression in ILD, based on sensitivity and accuracy?* | 207 | Very important | 48.8% | Median = 2  IQR = 1 | Yes |
|  |  | Important | 34.3% |  |  |
|  |  | Somewhat important | 15.0% |  |  |
|  |  | Neutral | 1.9% |  |  |
|  |  | Slightly not important | 0% |  |  |
|  |  | Not important | 0% |  |  |
|  |  | Not important at all | 0% |  |  |
| In normal clinical practice, how important is **decline in FVC** in monitoring for progression in ILD, based on sensitivity and accuracy?* | 207 | Very important | 76.3% | Median = 3  IQR = 0 | Yes |
|  |  | Important | 18.8% |  |  |
|  |  | Somewhat important | 4.4% |  |  |
|  |  | Neutral | 0.5% |  |  |
|  |  | Slightly not important | 0% |  |  |
|  |  | Not important | 0% |  |  |
|  |  | Not important at all | 0% |  |  |
| In normal clinical practice, how important is **decreased 6MWD** in monitoring for progression in ILD, based on sensitivity and accuracy?* | 207 | Very important | 27.1% | Median = 3  IQR = 0 | Yes |
|  |  | Important | 42.0% |  |  |
|  |  | Somewhat important | 28.0% |  |  |
|  |  | Neutral | 1.9% |  |  |
|  |  | Slightly not important | 0.5% |  |  |
|  |  | Not important | 0% |  |  |
|  |  | Not important at all | 0.5% |  |  |
| In normal clinical practice, how important is decreased **maximum exercise capacity** in monitoring for progression in ILD, based on sensitivity and accuracy?* | 207 | Very important | 8.2% | Median = 1  IQR = 0 | No |
|  |  | Important | 35.8% |  |  |
|  |  | Somewhat important | 33.8% |  |  |
|  |  | Neutral | 17.4% |  |  |
|  |  | Slightly not important | 1.5% |  |  |
|  |  | Not important | 2.4% |  |  |
|  |  | Not important at all | 1.0% |  |  |
| In normal clinical practice, how important is decreased **quality of life** in monitoring for progression in ILD, based on sensitivity and accuracy?* | 207 | Very important | 13.5% | Median = 1  IQR = 0 | No |
|  |  | Important | 37.2% |  |  |
|  |  | Somewhat important | 32.9% |  |  |
|  |  | Neutral | 11.6% |  |  |
|  |  | Slightly not important | 3.9% |  |  |
|  |  | Not important | 0.5% |  |  |
|  |  | Not important at all | 0.5% |  |  |
| In normal clinical practice, how important is decreased **total lung volume** in monitoring for progression in ILD, based on sensitivity and accuracy?* | 207 | Very important | 15.5% | Median = 1  IQR = 0 | No |
|  |  | Important | 35.8% |  |  |
|  |  | Somewhat important | 24.2% |  |  |
|  |  | Neutral | 16.9% |  |  |
|  |  | Slightly not important | 4.8% |  |  |
|  |  | Not important | 2.4% |  |  |
|  |  | Not important at all | 0.5% |  |  |
| In normal clinical practice, how important is **increase or initiation of** **supplemental oxygen** in monitoring for progression in ILD, based on sensitivity and accuracy?* | 207 | Very important | 42.0% | Median = 1  IQR = 1 | Yes |
|  |  | Important | 41.6% |  |  |
|  |  | Somewhat important | 14.0% |  |  |
|  |  | Neutral | 2.4% |  |  |
|  |  | Slightly not important | 0% |  |  |
|  |  | Not important | 0% |  |  |
|  |  | Not important at all | 0% |  |  |
| In normal clinical practice, how important is **increased fibrosis on HRCT** in monitoring for progression in ILD, based on sensitivity and accuracy?* | 207 | Very important | 60.9% | Median = 3  IQR = 1 | Yes |
|  |  | Important | 31.9% |  |  |
|  |  | Somewhat important | 6.3% |  |  |
|  |  | Neutral | 0.5% |  |  |
|  |  | Slightly not important | 0.5% |  |  |
|  |  | Not important | 0% |  |  |
|  |  | Not important at all | 0% |  |  |
| In normal clinical practice, how important is **nadir oxygen saturation on 6MWT** in monitoring for progression in ILD, based on sensitivity and accuracy?* | 207 | Very important | 17.4% | Median = 2  IQR = 1 | Yes |
|  |  | Important | 48.3% |  |  |
|  |  | Somewhat important | 24.6% |  |  |
|  |  | Neutral | 7.3% |  |  |
|  |  | Slightly not important | 1.5% |  |  |
|  |  | Not important | 0.5% |  |  |
|  |  | Not important at all | 0.5% |  |  |
| In normal clinical practice, how important is **onset of pulmonary hypertension** in monitoring for progression in ILD, based on sensitivity and accuracy?* | 207 | Very important | 30.9% | Median = 2  IQR = 2 | No |
|  |  | Important | 38.2% |  |  |
|  |  | Somewhat important | 25.1% |  |  |
|  |  | Neutral | 5.3% |  |  |
|  |  | Slightly not important | 0.5% |  |  |
|  |  | Not important | 0% |  |  |
|  |  | Not important at all | 0% |  |  |
| In normal clinical practice, how important is **respiratory hospitalisation** in monitoring for progression in ILD, based on sensitivity and accuracy?* | 207 | Very important | 31.4% | Median = 2  IQR = 2 | No |
|  |  | Important | 33.8% |  |  |
|  |  | Somewhat important | 23.2% |  |  |
|  |  | Neutral | 9.2% |  |  |
|  |  | Slightly not important | 1.0% |  |  |
|  |  | Not important | 1.0% |  |  |
|  |  | Not important at all | 0.5% |  |  |
| In normal clinical practice, how important is **weight loss** in monitoring for progression in ILD, based on sensitivity and accuracy?* | 207 | Very important | 6.3% | Median = 1  IQR = 2 | No |
|  |  | Important | 30.4% |  |  |
|  |  | Somewhat important | 31.4% |  |  |
|  |  | Neutral | 22.7% |  |  |
|  |  | Slightly not important | 6.3% |  |  |
|  |  | Not important | 1.0% |  |  |
|  |  | Not important at all | 1.9% |  |  |
| In normal clinical practice, how important is **worsening symptoms** in monitoring for progression in ILD, based on sensitivity and accuracy?* | 207 | Very important | 35.3% | Median = 2  IQR = 1 | Yes |
|  |  | Important | 46.9% |  |  |
|  |  | Somewhat important | 14.0% |  |  |
|  |  | Neutral | 2.4% |  |  |
|  |  | Slightly not important | 1.5% |  |  |
|  |  | Not important | 0% |  |  |
|  |  | Not important at all | 0% |  |  |
| How important are **quality of life measures and/or patient-reported outcomes** for routine monitoring for progression in your patients with ILD?* | 207 | Very important | 15.9% | Median = 2  IQR = 1 | Yes |
|  |  | Important | 44.9% |  |  |
|  |  | Somewhat important | 18.4% |  |  |
|  |  | Neutral | 12.1% |  |  |
|  |  | Slightly not important | 4.4% |  |  |
|  |  | Not important | 2.9% |  |  |
|  |  | Not important at all | 1.5% |  |  |
| In general, how often do you perform FVC measurements when following up with a patient with ILD? | 207 | Every time you see the patient | 65.7% | n/a | No |
|  |  | Not every time (please define frequency) | 33.8% |  |  |
|  |  | 2–6 months | 63.2% |  |  |
|  |  | 6–12 months | 29.7% |  |  |
|  |  | Unsure/not applicable | 0.5% |  |  |
| In general, how often do you perform DLco measurements when following up with a patient with ILD? | 207 | Every time you see the patient | 52.7% | n/a | No |
|  |  | Not every time (please define frequency) | 45.4% |  |  |
|  |  | 3–6 months | 40.6% |  |  |
|  |  | 3–12 months | 49.2% |  |  |
|  |  | Unsure/not applicable | 1.9% |  |  |
| In general, how often do you order a chest HRCT when following up with a patient with ILD^†^ | 207 | Per local protocol at defined time intervals | 8.2% | n/a | No |
|  |  | As clinically indicated, based on case-by-case evaluation | 64.7% |  |  |
|  |  | Both option 1 and option 2 | 33.8% |  |  |
|  |  | Unsure/not applicable | 1.0% |  |  |
| In general, how often do you perform a 6MWT when following up with a patient with ILD? | 207 | Every time you see the patient | 19.2% | n/a | No |
|  |  | Not every time [please define frequency] | 37.4% |  |  |
|  |  | 6–12 months | 35.7% |  |  |
|  |  | When additional information is required in order to identify progression | 39.9% |  |  |
|  |  | Unsure/not applicable | 3.5% |  |  |
| What are the real-world barriers when monitoring for progression in ILD?^†^ | 207 | Burden to the patients of repeated investigations and hospital visits | 58.0% | n/a | No |
|  |  | Cost of diagnostic tests | 43.0% |  |  |
|  |  | Access of patients to expert centres | 41.6% |  |  |
|  |  | Access to PFT labs | 38.2% |  |  |
|  |  | Radiation exposure | 27.5% |  |  |
|  |  | Patient reluctance or fear of testing | 27.1% |  |  |
|  |  | Continuity of care with patients | 26.1% |  |  |
|  |  | Access to CT scans | 25.6% |  |  |
|  |  | Unsure/not applicable | 6.3% |  |  |
|  |  | Other (please specify) | 14.5% |  |  |

*Statements were assessed on a 7-point Likert scale. The scale was from -3 (not important at all/strongly disagree) to +3 (very important/strongly agree). Data are % of respondents that selected each Likert scale category.

^†^More than one answer could be selected.

6MWD, six-minute walk distance; 6MWT, six-minute walk test; CT, computed tomography; DLco, diffusing capacity of the lungs for carbon monoxide; FVC, forced vital capacity; HRCT, high-resolution for computed tomography; ILD, interstitial lung disease; PFT, pulmonary function test.

**B**

| **Question/consensus statement** | **n** | **Response** | **% of respondents** | **Median and IQR for responses on Likert scale** | **Consensus?** |
| --- | --- | --- | --- | --- | --- |
| One or more occurrence of **acute exacerbations** with/without respiratory hospitalisation after diagnosis is an indicator of disease progression of fibrotic ILD in the absence of an alternative cause* | 131 | Strongly agree | 20.6% | Median = 2  IQR = 1 | Yes |
|  |  | Agree | 37.4% |  |  |
|  |  | Somewhat agree | 20.6% |  |  |
|  |  | Neutral | 9.9% |  |  |
|  |  | Somewhat disagree | 3.8% |  |  |
|  |  | Disagree | 6.1% |  |  |
|  |  | Strongly disagree | 1.5% |  |  |
| A **decline in DLco of 10–15**% from baseline is an indicator of disease progression of fibrotic ILD in the absence of an alternative cause* | 131 | Strongly agree | 18.3% | Median = 2  IQR = 1 | Yes |
|  |  | Agree | 48.9% |  |  |
|  |  | Somewhat agree | 24.4% |  |  |
|  |  | Neutral | 2.3% |  |  |
|  |  | Somewhat disagree | 3.8% |  |  |
|  |  | Disagree | 1.5% |  |  |
|  |  | Strongly disagree | 0.8% |  |  |
| A **decline in FVC of 10%** from baseline is an indicator of disease progression of fibrotic ILD in the absence of an alternative cause* | 131 | Strongly agree | 32.1% | Median = 2  IQR = 1 | Yes |
|  |  | Agree | 51.9% |  |  |
|  |  | Somewhat agree | 14.5% |  |  |
|  |  | Neutral | 0% |  |  |
|  |  | Somewhat disagree | 1.5% |  |  |
|  |  | Disagree | 0% |  |  |
|  |  | Strongly disagree | 0% |  |  |
| A **decline in 6MWD of 10–20%** from baseline is an indicator of disease progression of fibrotic ILD in the absence of an alternative cause* | 131 | Strongly agree | 12.2% | Median = 1  IQR = 1 | No |
|  |  | Agree | 35.9% |  |  |
|  |  | Somewhat agree | 32.8% |  |  |
|  |  | Neutral | 12.2% |  |  |
|  |  | Somewhat disagree | 4.6% |  |  |
|  |  | Disagree | 1.5% |  |  |
|  |  | Strongly disagree | 0.8% |  |  |
| A **decline in decreased maximum exercise capacity of 10–15%** from baseline is an indicator of disease progression of fibrotic ILD in the absence of an alternative cause* | 131 | Strongly agree | 9.2% | Median = 1  IQR = 1 | No |
|  |  | Agree | 33.6% |  |  |
|  |  | Somewhat agree | 35.9% |  |  |
|  |  | Neutral | 17.6% |  |  |
|  |  | Somewhat disagree | 2.3% |  |  |
|  |  | Disagree | 0.8% |  |  |
|  |  | Strongly disagree | 0.8% |  |  |
| A **decline in quality of life** from baseline is an indicator of disease progression of fibrotic ILD in the absence of an alternative cause* | 131 | Strongly agree | 4.6% | Median = 1  IQR = 2 | No |
|  |  | Agree | 22.1% |  |  |
|  |  | Somewhat agree | 35.1% |  |  |
|  |  | Neutral | 19.9% |  |  |
|  |  | Somewhat disagree | 13.7% |  |  |
|  |  | Disagree | 3.1% |  |  |
|  |  | Strongly disagree | 1.5% |  |  |
| A **decline in total lung volume of 10%** from baseline is an indicator of disease progression of fibrotic ILD in the absence of an alternative cause* | 131 | Strongly agree | 6.9% | Median = 0  IQR = 2 | No |
|  |  | Agree | 38.9% |  |  |
|  |  | Somewhat agree | 30.5% |  |  |
|  |  | Neutral | 16.8% |  |  |
|  |  | Somewhat disagree | 4.6% |  |  |
|  |  | Disagree | 2.3% |  |  |
|  |  | Strongly disagree | 0% |  |  |
| If CT is used to identify clinically significant disease progression, any serial changes on CT, however minor, are sufficient* | 131 | Strongly agree | 3.8% | Median = 0  IQR = 2 | No |
|  |  | Agree | 16.0% |  |  |
|  |  | Somewhat agree | 24.4% |  |  |
|  |  | Neutral | 12.2% |  |  |
|  |  | Somewhat disagree | 26.0% |  |  |
|  |  | Disagree | 16.0% |  |  |
|  |  | Strongly disagree | 1.5% |  |  |
| If CT is used to identify clinically significant disease progression, judgement should be made, case by case, on whether change on CT is convincing (i.e. occurs over multiple CT sections or is striking in a single CT section)* | 131 | Strongly agree | 32.8% | Median = 2  IQR = 1 | Yes |
|  |  | Agree | 49.6% |  |  |
|  |  | Somewhat agree | 13.7% |  |  |
|  |  | Neutral | 2.3% |  |  |
|  |  | Somewhat disagree | 0% |  |  |
|  |  | Disagree | 1.5% |  |  |
|  |  | Strongly disagree | 0% |  |  |
| **Weight loss of 5–10%** over a minimum of 3–6 months from baseline is an indicator of disease progression of fibrotic ILD in the absence of an alternative cause* | 131 | Strongly agree | 4.6% | Median = 0  IQR = 2 | No |
|  |  | Agree | 13.0% |  |  |
|  |  | Somewhat agree | 25.2% |  |  |
|  |  | Neutral | 25.2% |  |  |
|  |  | Somewhat disagree | 18.3% |  |  |
|  |  | Disagree | 10.7% |  |  |
|  |  | Strongly disagree | 3.1% |  |  |
| **Worsening of cough** from baseline is an indicator of disease progression of fibrotic ILD in the absence of an alternative cause* | 131 | Strongly agree | 4.6% | Median = 1  IQR = 2 | No |
|  |  | Agree | 24.4% |  |  |
|  |  | Somewhat agree | 33.6% |  |  |
|  |  | Neutral | 14.5% |  |  |
|  |  | Somewhat disagree | 13.0% |  |  |
|  |  | Disagree | 8.4% |  |  |
|  |  | Strongly disagree | 1.5% |  |  |
| **Worsening of dyspnoea** from baseline is an indicator of disease progression of fibrotic ILD in the absence of an alternative cause* | 131 | Strongly agree | 13.7% | Median = 2  IQR = 1 | Yes |
|  |  | Agree | 53.4% |  |  |
|  |  | Somewhat agree | 23.7% |  |  |
|  |  | Neutral | 3.1% |  |  |
|  |  | Somewhat disagree | 5.3% |  |  |
|  |  | Disagree | 0.8% |  |  |
|  |  | Strongly disagree | 0% |  |  |
| **A decline from baseline of respiratory health status** according to clinical judgement is an indicator of disease progression of fibrotic ILD in the absence of an alternative cause* | 131 | Strongly agree | 9.2% | Median = 1  IQR = 1 | No |
|  |  | Agree | 31.3% |  |  |
|  |  | Somewhat agree | 38.9% |  |  |
|  |  | Neutral | 13.0% |  |  |
|  |  | Somewhat disagree | 5.3% |  |  |
|  |  | Disagree | 1.5% |  |  |
|  |  | Strongly disagree | 0.8% |  |  |
| In general, you follow up with a patient more frequently depending on their risk of progression at baseline* | 131 | Strongly agree | 34.4% | Median = 2  IQR = 1 | Yes |
|  |  | Agree | 48.1% |  |  |
|  |  | Somewhat agree | 13.0% |  |  |
|  |  | Neutral | 3.1% |  |  |
|  |  | Somewhat disagree | 0% |  |  |
|  |  | Disagree | 0.8% |  |  |
|  |  | Strongly disagree | 0.8% |  |  |
| In general, in your individual practice, what is the most frequent time interval for how often FVC measurements are performed? | 131 | 3–6 months | 76.3% | n/a | Yes |
|  |  | 6–9 months | 19.9% |  |  |
|  |  | 9–12 months | 3.8% |  |  |
| In general, in your individual practice, what is the most frequent time interval for how often DLco measurements are performed? | 131 | 3–6 months | 64.1% | n/a | No |
|  |  | 6–9 months | 21.4% |  |  |
|  |  | 9–12 months | 14.5% |  |  |
| A 6MWT does not need to be performed every time you see the patient; it should be performed as needed (e.g. when additional information is required in order to identify progression)* | 131 | Strongly agree | 13.7% | Median = 2  IQR = 1.5 | No |
|  |  | Agree | 45.0% |  |  |
|  |  | Somewhat agree | 16.0% |  |  |
|  |  | Neutral | 5.3% |  |  |
|  |  | Somewhat disagree | 7.6% |  |  |
|  |  | Disagree | 8.4% |  |  |
|  |  | Strongly disagree | 3.8% |  |  |
| In clinical practice, progression of ILD can be identified with lesser declines than those thresholds specified above, provided that trends are consistent across many variables* | 131 | Strongly agree | 16.8% | Median = 2  IQR = 1 | Yes |
|  |  | Agree | 42.8% |  |  |
|  |  | Somewhat agree | 24.4% |  |  |
|  |  | Neutral | 8.4% |  |  |
|  |  | Somewhat disagree | 3.8% |  |  |
|  |  | Disagree | 3.8% |  |  |
|  |  | Strongly disagree | 0% |  |  |

*Statements were assessed on a 7-point Likert scale. The scale was from -3 (not important at all/strongly disagree) to +3 (very important/strongly agree). Data are % of respondents that selected each Likert scale category.

^†^More than one answer could be selected.

6MWD, six-minute walk distance; 6MWT, six-minute walk test; CT, computed tomography; DLco, diffusing capacity of the lungs for carbon monoxide; FVC, forced vital capacity; HRCT, high-resolution for computed tomography; ILD, interstitial lung disease; PFT, pulmonary function test.

**C**

| **Question/consensus statement** | **n** | **Response** | **% of respondents** | **Median and IQR for responses on Likert scale** | **Consensus?** |
| --- | --- | --- | --- | --- | --- |
| To what extent do you agree with the following: “Where possible, **6MWD** should be monitored, as a decline from baseline may serve as an indicator of disease progression in fibrotic ILD”?* | 94 | Strongly agree | 13.8% | Median = 2  IQR = 0.75 | Yes |
|  |  | Agree | 60.6% |  |  |
|  |  | Somewhat agree | 21.3% |  |  |
|  |  | Neutral | 3.2% |  |  |
|  |  | Somewhat disagree | 0% |  |  |
|  |  | Disagree | 1.1% |  |  |
|  |  | Strongly disagree | 0% |  |  |
| To what extent do you agree with the following: “Where possible, **maximum exercise capacity** should be monitored, as a decline from baseline may serve as an indicator of disease progression in fibrotic ILD”?* | 94 | Strongly agree | 7.5% | Median = 2  IQR = 1 | Yes |
|  |  | Agree | 46.8% |  |  |
|  |  | Somewhat agree | 27.7% |  |  |
|  |  | Neutral | 10.6% |  |  |
|  |  | Somewhat disagree | 4.3% |  |  |
|  |  | Disagree | 2.1% |  |  |
|  |  | Strongly disagree | 1.1% |  |  |
| To what extent do you agree with the following: “Where possible, **total lung volume** should be monitored, as a decline from baseline may serve as an indicator of disease progression in patients with fibrotic ILD in the absence of an alternative cause”?* | 94 | Strongly agree | 19.2% | Median = 2  IQR = 1 | Yes |
|  |  | Agree | 44.7% |  |  |
|  |  | Somewhat agree | 22.3% |  |  |
|  |  | Neutral | 8.5% |  |  |
|  |  | Somewhat disagree | 3.2% |  |  |
|  |  | Disagree | 1.1% |  |  |
|  |  | Strongly disagree | 1.1% |  |  |
| To what extent do you agree with the following: “The occurrence of otherwise unexplained weight loss should prompt an evaluation for disease progression in patients with fibrotic ILD”?* | 94 | Strongly agree | 4.3% | Median = 1  IQR = 2 | No |
|  |  | Agree | 43.6% |  |  |
|  |  | Somewhat agree | 22.3% |  |  |
|  |  | Neutral | 14.9% |  |  |
|  |  | Somewhat disagree | 9.6% |  |  |
|  |  | Disagree | 5.3% |  |  |
|  |  | Strongly disagree | 0% |  |  |
| To what extent do you agree with the following: “The occurrence of otherwise **unexplained worsening cough** should prompt an evaluation for disease progression in patients with fibrotic ILD”?* | 94 | Strongly agree | 5.3% | Median = 2  IQR = 1 | Yes |
|  |  | Agree | 64.9% |  |  |
|  |  | Somewhat agree | 17.0% |  |  |
|  |  | Neutral | 7.5% |  |  |
|  |  | Somewhat disagree | 3.2% |  |  |
|  |  | Disagree | 2.1% |  |  |
|  |  | Strongly disagree | 0% |  |  |
| To what extent do you agree with the following: “Any otherwise unexplained **decline in respiratory-health status** should prompt an evaluation of disease progression in patients with fibrotic ILD”?* | 94 | Strongly agree | 13.8% | Median = 2  IQR = 1 | Yes |
|  |  | Agree | 56.4% |  |  |
|  |  | Somewhat agree | 19.2% |  |  |
|  |  | Neutral | 10.6% |  |  |
|  |  | Somewhat disagree | 0% |  |  |
|  |  | Disagree | 0% |  |  |
|  |  | Strongly disagree | 0% |  |  |
| To what extent do you agree with the following: “Where possible, DLco measurements should be performed every 6±3 months”?* | 94 | Strongly agree | 40.4% | Median = 2  IQR = 1 | Yes |
|  |  | Agree | 44.7% |  |  |
|  |  | Somewhat agree | 7.5% |  |  |
|  |  | Neutral | 4.3% |  |  |
|  |  | Somewhat disagree | 1.1% |  |  |
|  |  | Disagree | 1.1% |  |  |
|  |  | Strongly disagree | 1.1% |  |  |
| To what extent do you agree with the following: “A 6MWT does not need to be performed every time you see the patient; if the test is available, it should be performed as needed (for example, it may provide additional information to support finding of progressive disease)”?* | 94 | Strongly agree | 17.0% | Median = 2  IQR = 0.75 | Yes |
|  |  | Agree | 57.5% |  |  |
|  |  | Somewhat agree | 8.5% |  |  |
|  |  | Neutral | 7.5% |  |  |
|  |  | Somewhat disagree | 3.2% |  |  |
|  |  | Disagree | 5.3% |  |  |
|  |  | Strongly disagree | 1.1% |  |  |

*Statements were assessed on a 7-point Likert scale. The scale was from -3 (not important at all/strongly disagree) to +3 (very important/strongly agree). Data are % of respondents that selected each Likert scale category.

^†^More than one answer could be selected.

6MWD, six-minute walk distance; 6MWT, six-minute walk test; CT, computed tomography; DLco, diffusing capacity of the lungs for carbon monoxide; FVC, forced vital capacity; HRCT, high-resolution for computed tomography; ILD, interstitial lung disease; PFT, pulmonary function test.

**Table S4** Questions and responses on “Defining progression in the real world” from **A**) survey 1, **B**) survey 2 and **C**) survey 3.

**A**

| **Question/consensus statement** | **n** | **Response** | **% of respondents** | **Median and IQR for responses on Likert scale** | **Consensus?** |
| --- | --- | --- | --- | --- | --- |
| ***Survey 1*** | | | | |  |
| There is a need to better define disease progression in ILD* | 207 | Strongly agree | 37.2% | Median = 2  IQR = 1 | Yes |
|  |  | Agree | 44.0% |  |  |
|  |  | Somewhat agree | 13.0% |  |  |
|  |  | Neutral | 2.4% |  |  |
|  |  | Somewhat disagree | 1.9% |  |  |
|  |  | Disagree | 0.5% |  |  |
|  |  | Strongly disagree | 1.0% |  |  |
| Should PF-ILDs be considered together as a group, or separately as individual diseases for assessing the risk of progression at the time of initial diagnosis? | 207 | Group together | 19.3% | n/a | Yes |
|  |  | As individual diseases | 80.7% |  |  |
| Should PF-ILDs be considered together as a group, or separately as individual diseases for assessing the risk of future progression once longitudinal progression is identified? | 207 | Group together | 42.5% | n/a | No |
|  |  | As individual diseases | 57.5% |  |  |
| Should PF-ILDs be considered together as a group, or separately as individual diseases for defining what clinical tools should be used to identify progression? | 207 | Group together | 62.3% | n/a | No |
|  |  | As individual diseases | 37.7% |  |  |
| Which ILDs would require distinct definitions of progression?^†^ | 207 | Sarcoidosis | 62.8% | n/a | No |
|  |  | SSc-ILD | 52.7% |  |  |
|  |  | IPF | 51.7% |  |  |
|  |  | Fibrotic HP | 47.8% |  |  |
|  |  | IPAF | 47.8% |  |  |
|  |  | RA-ILD | 47.3% |  |  |
|  |  | Myositis (polymyositis, dermatomyositis) | 45.4% |  |  |
|  |  | PPFE | 44.4% |  |  |
|  |  | Post-infection ILD | 43.5% |  |  |
|  |  | Unclassifiable ILD/IIP | 43.5% |  |  |
|  |  | Mixed CTD-ILD | 42.0% |  |  |
|  |  | Sjögren’s disease | 41.1% |  |  |
|  |  | Idiopathic fibrosing NSIP | 40.1% |  |  |
|  |  | Genetic ILD (e.g. Hermansky-Pudlak syndrome, short telomere, etc.) | 38.2% |  |  |
|  |  | Pneumoconiosis | 37.7% |  |  |
|  |  | Other (please specify) | 13.0% |  |  |
| In the absence of an alternative explanation, is an isolated decline in FVC beyond a defined threshold (in an agreed timeframe) sufficient to determine progression of lung fibrosis? | 207 | Yes | 64.3% | n/a | No |
|  |  | No | 35.8% |  |  |
| In the absence of an alternative explanation, is an isolated deterioration in DLco beyond a defined threshold (in an agreed timeframe) sufficient to determine progression of lung fibrosis? | 207 | Yes | 54.6% | n/a | No |
|  |  | No | 45.4% |  |  |
| In the absence of an alternative explanation, is progression on HRCT (in an agreed timeframe) sufficient to determine progression of lung fibrosis? | 207 | Yes | 74.4% | n/a | Yes |
|  |  | No | 25.6% |  |  |
| In the absence of an alternative explanation, is an isolated decline in 6MWD beyond a defined threshold (in an agreed timeframe) sufficient to determine progression of lung fibrosis? | 207 | Yes | 39.6% | n/a | No |
|  |  | No | 60.4% |  |  |
| In the absence of an alternative explanation, is worsening of respiratory symptoms (in an agreed timeframe) sufficient to determine progression of lung fibrosis? | 207 | Yes | 38.2% | n/a | No |
|  |  | No | 61.8% |  |  |
| Does a small decline in multiple endpoints (in an agreed timeframe) indicate progression? For example, smaller changes in FVC combined with small changes in 6MWD | 207 | Yes | 70.0% | n/a | Yes |
|  |  | No | 30.0% |  |  |
| When considering a decline as determining progression, is an isolated decline at a single time point sufficient or is a trend required for confirmation? For example, if a patient’s FVC declines at a given time point, do you need to see that decline maintained at the next visit to consider it progression? | 207 | Single endpoint: Single | 16.9% | n/a | Yes |
|  |  | Single endpoint: Trend | 83.1% |  |  |
|  | 207 | For multiple endpoints: Single | 54.6% |  | No |
|  |  | For multiple endpoints: Trend | 45.4% |  |  |
|  | 207 | For changes below a threshold: Single | 31.9% |  | No |
|  |  | For changes below a threshold: Trend | 68.1% |  |  |
| Which endpoints can be used to define progression? Please rank the endpoints in order of importance in determining progression of fibrotic lung disease | 207 | Ranking from most to least important^‡^: | n/a | Median ranking/score | n/a |
|  |  | Decline in FVC |  | 2.0 |  |
|  |  | Decline in DLco |  | 3.0 |  |
|  |  | Increased fibrosis on HRCT |  | 4.0 |  |
|  |  | Acute exacerbation |  | 5.0 |  |
|  |  | Decreased 6MWD |  | 6.0 |  |
|  |  | Worsening symptoms |  | 6.0 |  |
|  |  | Increase or initiation of supplemental oxygen |  | 7.0 |  |
|  |  | Decreased maximum exercise capacity |  | 9.0 |  |
|  |  | Decreased total lung volume |  | 9.0 |  |
|  |  | Nadir oxygen saturation on 6MWD |  | 9.0 |  |
|  |  | Decreased quality of life |  | 10.0 |  |
|  |  | Onset of pulmonary hypertension |  | 10.0 |  |
|  |  | Respiratory hospitalisation |  | 11.0 |  |
|  |  | Weight loss |  | 13.0 |  |

*Statements were assessed on a 7-point Likert scale. The scale was from -3 (strongly disagree) to +3 (strongly agree). Data are % of respondents that selected each Likert scale category.

^†^More than one answer could be selected.

^‡^Data shown are median ranking/score.

6MWD, six-minute walk distance; CTD-ILD, connective tissue disease-associated interstitial lung disease; DLco, diffusing capacity of the lungs for carbon monoxide; FVC, forced vital capacity; HRCT, high-resolution for computed tomography; HP, hypersensitivity pneumonitis; IIP, idiopathic interstitial pneumonia; ILD, interstitial lung disease; IPAF, interstitial pneumonia with autoimmune features; IPF, idiopathic pulmonary fibrosis; NSIP, non-specific interstitial pneumonia; PF-ILD, progressive fibrosis interstitial lung disease; RA-ILD, rheumatoid arthritis-associated interstitial lung disease; SSc-ILD scleroderma-associated interstitial lung disease.

**B**

| **Question/consensus statement** | **n** | **Response** | **% of respondents** | **Median and IQR for responses on Likert scale** | **Consensus?** |
| --- | --- | --- | --- | --- | --- |
| Progression after 12 months since diagnosis should be considered as progression* | 131 | Strongly agree | 35.1% | Median = 2  IQR = 1 | Yes |
|  |  | Agree | 48.9% |  |  |
|  |  | Somewhat agree | 9.2% |  |  |
|  |  | Neutral | 1.5% |  |  |
|  |  | Somewhat disagree | 3.1% |  |  |
|  |  | Disagree | 1.5% |  |  |
|  |  | Strongly disagree | 0.8% |  |  |
| Progression of ILD is progression irrespective of time since diagnosis (although impact on prognosis management may depend on timelines)* | 131 | Strongly agree | 24.4% | Median = 2  IQR = 1 | Yes |
|  |  | Agree | 37.4% |  |  |
|  |  | Somewhat agree | 21.4% |  |  |
|  |  | Neutral | 4.6% |  |  |
|  |  | Somewhat disagree | 7.6% |  |  |
|  |  | Disagree | 3.1% |  |  |
|  |  | Strongly disagree | 1.5% |  |  |
| If defining progression of ILD does depend on time since diagnosis, what time parameter would you use?^‡^ | 16 | 12 months | 62.5% | n/a | No |
|  |  | 18 months | 0% |  |  |
|  |  | 24 months | 37.5% |  |  |
|  |  | 36 months | 0% |  |  |
| In general, there is a minimum time period for progression of ILD* | 131 | Strongly agree | 9.9% | Median = 0  IQR = 3.5 | No |
|  |  | Agree | 26.7% |  |  |
|  |  | Somewhat agree | 19.1% |  |  |
|  |  | Neutral | 2.3% |  |  |
|  |  | Somewhat disagree | 16.0% |  |  |
|  |  | Disagree | 19.9% |  |  |
|  |  | Strongly disagree | 6.1% |  |  |
| In general, there is a maximum time period for progression of ILD* | 131 | Strongly agree | 4.6% | Median = -1  IQR = 3 | No |
|  |  | Agree | 15.3% |  |  |
|  |  | Somewhat agree | 10.7% |  |  |
|  |  | Neutral | 11.5% |  |  |
|  |  | Somewhat disagree | 22.9% |  |  |
|  |  | Disagree | 27.5% |  |  |
|  |  | Strongly disagree | 7.6% |  |  |
| In general, there is a maximum period of time you would monitor a patient with ILD before deciding that the disease is ‘not progressing’* | 131 | Strongly agree | 6.1% | Median = 1  IQR = 4 | No |
|  |  | Agree | 23.7% |  |  |
|  |  | Somewhat agree | 16.8% |  |  |
|  |  | Neutral | 10.7% |  |  |
|  |  | Somewhat disagree | 17.6% |  |  |
|  |  | Disagree | 22.1% |  |  |
|  |  | Strongly disagree | 3.1% |  |  |
| ILD progression should be defined differently for individual diseases* | 131 | Strongly agree | 11.5% | Median = 1  IQR = 3 | No |
|  |  | Agree | 22.9% |  |  |
|  |  | Somewhat agree | 20.6% |  |  |
|  |  | Neutral | 13.0% |  |  |
|  |  | Somewhat disagree | 11.5% |  |  |
|  |  | Disagree | 18.3% |  |  |
|  |  | Strongly disagree | 2.3% |  |  |
| In general, what factors define progression in **idiopathic pulmonary fibrosis**, assuming sustained decline in the absence of alternative explanations?^†^ | 131 | Increased fibrosis on HRCT | 97.0% | n/a | Yes |
|  |  | Decline in FVC | 97.0% |  |  |
|  |  | Decline in DLco | 89.3% |  |  |
|  |  | Worsening of respiratory symptoms | 87.0% |  |  |
|  |  | Deterioration in 6MWD | 74.8% |  |  |
|  |  | Acute exacerbation | 68.7% |  |  |
| In general, what factors define progression in **systemic sclerosis-associated ILD**, assuming sustained decline in the absence of alternative explanations?^†^ | 131 | Increased fibrosis on HRCT | 98.5% | n/a | Yes |
|  |  | Decline in FVC | 97.7% |  |  |
|  |  | Decline in DLco | 88.6% |  |  |
|  |  | Worsening of respiratory symptoms | 81.7% |  |  |
|  |  | Deterioration in 6MWD | 61.8% |  |  |
|  |  | Acute exacerbation | 53.4% |  |  |
| In general, what factors define progression in **sarcoidosis**, assuming sustained decline in the absence of alternative explanations?^†^ | 131 | Increased fibrosis on HRCT | 96.2% | n/a | Yes |
|  |  | Decline in FVC | 90.1% |  |  |
|  |  | Decline in DLco | 86.3% |  |  |
|  |  | Worsening of respiratory symptoms | 77.9% |  |  |
|  |  | Deterioration in 6MWD | 60.3% |  |  |
|  |  | Acute exacerbation | 38.2% |  |  |
| In general, what factors define progression in **fibrotic HP**, assuming sustained decline in the absence of alternative explanations?^†^ | 131 | Increased fibrosis on HRCT | 98.5% | n/a | Yes |
|  |  | Decline in FVC | 96.2% |  |  |
|  |  | Decline in DLco | 87.8% |  |  |
|  |  | Worsening of respiratory symptoms | 83.2% |  |  |
|  |  | Deterioration in 6MWD | 65.7% |  |  |
|  |  | Acute exacerbation | 59.5% |  |  |
| In general, what factors define progression in **rheumatoid arthritis-ILD**, assuming sustained decline in the absence of alternative explanations?^†^ | 131 | Increased fibrosis on HRCT | 97.7% | n/a | Yes |
|  |  | Decline in FVC | 96.2% |  |  |
|  |  | Decline in DLco | 86.3% |  |  |
|  |  | Worsening of respiratory symptoms | 82.4% |  |  |
|  |  | Deterioration in 6MWD | 60.3% |  |  |
|  |  | Acute exacerbation | 57.3% |  |  |
| In general, what factors define progression in **myositis (polymyositis, dermatomyositis),** assuming sustained decline in the absence of alternative explanations?^†^ | 131 | Increased fibrosis on HRCT | 94.7% | n/a | Yes |
|  |  | Decline in FVC | 93.1% |  |  |
|  |  | Decline in DLco | 85.5% |  |  |
|  |  | Worsening of respiratory symptoms | 79.4% |  |  |
|  |  | Deterioration in 6MWD | 57.3% |  |  |
|  |  | Acute exacerbation | 55.0% |  |  |
| In the absence of an alternative explanation, ≥10% decline from baseline in FVC is sufficient to determine progression of pulmonary fibrosis* | 131 | Strongly agree | 21.4% | Median = 2  IQR = 1 | Yes |
|  |  | Agree | 45.0% |  |  |
|  |  | Somewhat agree | 19.9% |  |  |
|  |  | Neutral | 2.3% |  |  |
|  |  | Somewhat disagree | 5.3% |  |  |
|  |  | Disagree | 4.6% |  |  |
|  |  | Strongly disagree | 1.5% |  |  |
| An isolated decline in DLco distinguishes between progressive ILD and progressive vasculopathy* | 131 | Strongly agree | 2.3% | Median = -1  IQR = 3 | No |
|  |  | Agree | 10.7% |  |  |
|  |  | Somewhat agree | 28.2% |  |  |
|  |  | Neutral | 6.9% |  |  |
|  |  | Somewhat disagree | 17.6% |  |  |
|  |  | Disagree | 22.1% |  |  |
|  |  | Strongly disagree | 12.2% |  |  |

*Statements were assessed on a 7-point Likert scale. The scale was from -3 (strongly disagree) to +3 (strongly agree). Data are % of respondents that selected each Likert scale category.

^†^More than one answer could be selected.

^‡^Data shown are median ranking/score.

6MWD, six-minute walk distance; CTD-ILD, connective tissue disease-associated interstitial lung disease; DLco, diffusing capacity of the lungs for carbon monoxide; FVC, forced vital capacity; HRCT, high-resolution for computed tomography; HP, hypersensitivity pneumonitis; IIP, idiopathic interstitial pneumonia; ILD, interstitial lung disease; IPAF, interstitial pneumonia with autoimmune features; IPF, idiopathic pulmonary fibrosis; NSIP, non-specific interstitial pneumonia; PF-ILD, progressive fibrosis interstitial lung disease; RA-ILD, rheumatoid arthritis-associated interstitial lung disease; SSc-ILD scleroderma-associated interstitial lung disease.

**C**

| **Question/consensus statement** | **n** | **Response** | **% of respondents** | **Median and IQR for responses on Likert scale** | **Consensus?** |
| --- | --- | --- | --- | --- | --- |
| To what extent do you agree with the following: “An isolated decline in DLco favours progressive vasculopathy over progressive ILD”?* | 94 | Strongly agree | 5.3% | Median = 1  IQR = 2 | No |
|  |  | Agree | 28.7% |  |  |
|  |  | Somewhat agree | 30.9% |  |  |
|  |  | Neutral | 13.8% |  |  |
|  |  | Somewhat disagree | 10.6% |  |  |
|  |  | Disagree | 9.6% |  |  |
|  |  | Strongly disagree | 1.1% |  |  |

*Statements were assessed on a 7-point Likert scale. The scale was from -3 (strongly disagree) to +3 (strongly agree). Data are % of respondents that selected each Likert scale category.

^†^More than one answer could be selected.

^‡^Data shown are median ranking/score.

6MWD, six-minute walk distance; CTD-ILD, connective tissue disease-associated interstitial lung disease; DLco, diffusing capacity of the lungs for carbon monoxide; FVC, forced vital capacity; HRCT, high-resolution for computed tomography; HP, hypersensitivity pneumonitis; IIP, idiopathic interstitial pneumonia; ILD, interstitial lung disease; IPAF, interstitial pneumonia with autoimmune features; IPF, idiopathic pulmonary fibrosis; NSIP, non-specific interstitial pneumonia; PF-ILD, progressive fibrosis interstitial lung disease; RA-ILD, rheumatoid arthritis-associated interstitial lung disease; SSc-ILD scleroderma-associated interstitial lung disease.

**Table S5** Responses to “What level of decline and time period can define progression?” in survey 1.

| Acute exacerbations with/without respiratory hospitalisation [only responses chosen by >2.5% of respondents are shown] | **N** | **Number of acute exacerbations** | **Time period** | | **% of respondents** | **Consensus?** |
| --- | --- | --- | --- | --- | --- | --- |
|  | 207 | ≥1 | 12 months | | 86% | Yes |
|  |  | 1 | 12 months | | 68% | No |
|  |  | 1 | 6 months | | 52% | No |
|  |  | ≥1 | 24 months | | 31% | No |
|  |  | ≥1 | 6 months | | 26% | No |
|  |  | 1 | 1 month | | 23% | No |
|  |  | 1 | any time | | 21% | No |
|  |  | 1 | 3 months | | 20% | No |
|  |  | ≥1 | Any | | 17% | No |
|  |  | ≥1 | 3 months | | 11% | No |
|  |  | ≥1 | 1 month | | 8% | No |
|  |  | 1 | 24 months | | 5% | No |
|  |  | ≥1 | 36 months | | 5% | No |
|  |  | ≥1 | 18 months | | 4% | No |
| DLco [only responses chosen by >2.5% of respondents are shown] | N | **Decline in DLco** | **Minimum time period (months)** | **Maximum time period (months)** | **% of respondents** | **Consensus?** |
|  | 207 | 15% | 6 | 12 | 13.5% | No |
|  |  | 10% | 6 | 12 | 11.1% | No |
|  |  | 15% | 3 | 12 | 6.3% | No |
|  |  | 10% | 3 | 6 | 4.8% | No |
|  |  | 15% | 6 | 24 | 4.8% | No |
|  |  | 10% | 3 | 12 | 3.4% | No |
|  |  | 15% | 12 | 24 | 3.4% | No |
|  |  | 5% | 6 | 12 | 2.9% | No |
|  |  | 15% | 3 | 6 | 2.9% | No |
| FVC [only responses chosen by >2.5% of participants are shown] | **N** | **Decline in FVC** | **Minimum time period (months)** | **Maximum time period (months)** | **% of respondents** | **Consensus?** |
|  | 207 | 10% | 6 | 12 | 18.8% | No |
|  |  | 10% | 3 | 6 | 9.2% | No |
|  |  | 10% | 3 | 12 | 8.2% | No |
|  |  | 5% | 6 | 12 | 4.8% | No |
|  |  | 10% | 6 | 24 | 4.8% | No |
|  |  | 10% | 12 | 12 | 3.9% | No |
|  |  | 10% | 3 | 24 | 2.9% | No |
|  |  | 10% | 12 | 24 | 2.9% | No |
| 6MWD [only responses chosen by >2.5% of respondents are shown] | **N** | **Decline in 6MWD** | **Minimum time period (months)** | **Maximum time period (months)** | **% of respondents** | **Consensus?** |
|  | 207 | 10% | 6 | 12 | 10.6% | No |
|  |  | 15% | 6 | 12 | 6.3% | No |
|  |  | 20% | 6 | 12 | 4.3% | No |
|  |  | 10% | 6 | 24 | 3.9% | No |
|  |  | 15% | 3 | 6 | 3.4% | No |
|  |  | 20% | 3 | 6 | 3.4% | No |
|  |  | 20% | 3 | 12 | 3.4% | No |
|  |  | 10% | 3 | 6 | 2.9% | No |
|  |  | 10% | 3 | 12 | 2.9% | No |
|  |  | 10% | 12 | 24 | 2.9% | No |
| Maximum exercise capacity [only responses chosen by >2.5% of respondents are shown] | **N** | **Decrease in maximum exercise capacity** | **Minimum time period (months)** | **Maximum time period (months)** | **% of respondents** | **Consensus?** |
|  | 207 | 10% | 6 | 12 | 12.6% | No |
|  |  | 10% | 3 | 6 | 5.3% | No |
|  |  | 15% | 6 | 12 | 5.3% | No |
|  |  | 10% | 3 | 12 | 4.8% | No |
|  |  | 10% | 6 | 24 | 4.3% | No |
|  |  | 10% | 12 | 24 | 4.3% | No |
|  |  | 20% | 6 | 12 | 4.3% | No |
|  |  | 20% | 3 | 6 | 3.9% | No |
|  |  | Unsure/NA | Unsure/NA | Unsure/NA | 3.9% | No |
|  |  | 20% | 3 | 12 | 3.4% | No |
|  |  | 25% | 6 | 12 | 2.9% | No |
| Quality of life [only responses chosen by >1.0% of respondents are shown] | **N** | **Decreased quality of life** | **Minimum time period (months)** | **Maximum time period (months)** | **% of respondents** | **Consensus?** |
|  | 207 | Unsure/NA | Unsure/NA | Unsure/NA | 4.8% | No |
|  |  | Yes | 6 | 12 | 3.4% | No |
|  |  | Unsure/NA | 6 | 12 | 2.9% | No |
|  |  | 15 | 6 | 12 | 2.4% | No |
|  |  | 20 | 6 | 12 | 2.4% | No |
|  |  | Decreased quality of life not based on score | 3 | 6 | 2.4% | No |
|  |  | Yes | 3 | 6 | 2.4% | No |
|  |  | 10 | 6 | 12 | 1.9% | No |
|  |  | Yes | 3 | 12 | 1.9% | No |
|  |  | 10% | 1 | 24 | 1.4% | No |
|  |  | 20% | 1 | 24 | 1.4% | No |
|  |  | >MCID | 6 | 12 | 1.4% | No |
|  |  | Yes | 12 | 24 | 1.4% | No |
| Total lung volume [only responses chosen by >2.5% of respondents are shown] | **N** | **Decreased total lung volume** | **Minimum time period (months)** | **Maximum time period (months)** | **% of respondents** | **Consensus?** |
|  | 207 | 10% | 6 | 12 | 16.4% | No |
|  |  | 10% | 3 | 6 | 6.8% | No |
|  |  | 10% | 3 | 12 | 5.8% | No |
|  |  | Unsure/NA | Unsure/NA | Unsure/NA | 5.8% | No |
|  |  | 20% | 6 | 12 | 4.3% | No |
|  |  | 10% | 12 | 24 | 3.4% | No |
|  |  | 10% | 3 | 24 | 2.9% | No |
|  |  | 10% | 6 | 24 | 2.9% | No |
|  |  | 10% | 12 | 12 | 2.9% | No |
|  |  | 15% | 3 | 6 | 2.9% | No |
|  |  | 15% | 6 | 12 | 2.9% | No |
| Fibrosis on HRCT [only responses chosen by >2.5% of respondents are shown] | **N** | **Increased fibrosis on HRCT** | **Minimum time period (months)** | **Maximum time period (months)** | **% of respondents** | **Consensus?** |
|  | 207 | 10% | 6 | 12 | 15.0% | No |
|  |  | 10% | 3 | 12 | 5.8% | No |
|  |  | 5% | 6 | 12 | 5.3% | No |
|  |  | 20% | 6 | 12 | 4.3% | No |
|  |  | 5% | 6 | 24 | 3.9% | No |
|  |  | 10% | 3 | 6 | 3.4% | No |
|  |  | 10% | 12 | 24 | 3.4% | No |
| Supplemental oxygen use [only responses chosen by >2.5% of respondents are shown] | **N** | **Initiation or increased use of supplemental oxygen** | **Minimum time period (months)** | **Maximum time period (months)** | **% of respondents** | **Consensus?** |
|  | 207 | Yes | 6 | 12 | 5.8% | No |
|  |  | 2 L/min | 6 | 12 | 5.8% | No |
|  |  | Yes | 6 | 12 | 5.3% | No |
|  |  | Yes | 3 | 12 | 4.3% | No |
|  |  | Yes | Any | Any | 2.9% | No |
|  |  | 1 L/min | 6 | 12 | 2.9% | No |
| Weight loss [[only responses chosen by >2.5% of respondents are shown] | **N** | **Weight loss** | **Minimum time period (months)** | **Maximum time period (months)** | **% of respondents** | **Consensus?** |
|  | 207 | 10% | 6 | 12 | 14.0% | No |
|  |  | 10% | 3 | 6 | 7.7% | No |
|  |  | 10% | 3 | 12 | 7.2% | No |
|  |  | 5% | 6 | 12 | 4.8% | No |
|  |  | 10% | 12 | 24 | 4.3% | No |
|  |  | Unsure/NA | Unsure/NA | Unsure/NA | 3.9% | No |
| Worsening symptoms [only responses chosen by >2.5% of respondents are shown] | **N** | **Worsening symptoms** | **Minimum time period (months)** | **Maximum time period (months)** | **% of respondents** | **Consensus?** |
|  | 207 | Cough and/or dyspnoea | 6 | 12 | 5.3% | No |
|  |  | Yes | 6 | 12 | 4.8% | No |
|  |  | Cough and/or dyspnoea | 3 | 6 | 4.3% | No |
|  |  | Cough and/or dyspnoea | 6 | 24 | 3.4% | No |
|  |  | Yes | 3 | 6 | 3.4% | No |
|  |  | Cough and/or dyspnoea | 3 | 12 | 2.9% | No |
| Clinical judgement [only responses chosen by >2.5% of respondents are shown] | **N** | **General clinical judgement/gestalt** | **Minimum time period (months)** | **Maximum time period (months)** | **% of respondents** | **Consensus?** |
|  | 207 | Yes | 6 | 12 | 6.3% | No |
|  |  | Yes | 3 | 6 | 2.9% | No |
|  |  | Yes | 3 | 12 | 2.9% | No |
|  |  | Unsure/NA | 6 | 12 | 2.4% | No |
|  |  | Unsure/NA | Unsure/NA | Unsure/NA | 2.4% | No |
|  |  | 1 | 3 | 6 | 1.9% | No |
|  |  | Yes | 12 | 24 | 1.9% | No |
| Other measures | **N** | **Measure of progression** | | | **% of respondents** | **Consensus?** |
|  | 207 | N/A or none | | | 51.5% | No |
|  |  | Development of hypertension | | | 8.8% | No |
|  |  | Other | | | 7.4% | No |
|  |  | Clinical worsening/symptoms | | | 5.9% | No |
|  |  | Death or hospitalisation | | | 5.9% | No |
|  |  | Combined endpoint | | | 4.4% | No |
|  |  | Cough measures | | | 4.4% | No |
|  |  | Oxygen saturation after exercise | | | 4.4% | No |
|  |  | Patient/family/caretaker perspectives | | | 4.4% | No |
|  |  | Biomarkers | | | 2.9% | No |

**Table S6** Questions and responses on “managing progression” in survey 1.

| **Question/consensus statement** | **n** | **Response** | **% of respondents** | **Consensus?** |
| --- | --- | --- | --- | --- |
| In general, what is your opinion on what “appropriate management” means for patients with ILD before they are considered as having progressive fibrosis? | 207 | Depends on the type of ILD | 14.7% | No |
|  |  | Evaluation | 10.9% |  |
|  |  | Antifibrotic or immunosuppression | 7.6% |  |
|  |  | Immunosuppression/steroids | 7.6% |  |
|  |  | Define diagnosis | 5.7% |  |
|  |  | Maximise treatment | 4.7% |  |
|  |  | Standard of care | 3.8% |  |
|  |  | Reduce symptoms | 3.3% |  |
|  |  | Guidelines | 2.8% |  |
|  |  | Multiple steps | 2.8% |  |
|  |  | Treat when progression/lack of response | 2.4% |  |
|  |  | To achieve stable outcome | 2.4% |  |
|  |  | Wait and watch | 0.5% |  |
|  |  | Early antifibrotic | 0.5% |  |
|  |  | N/A | 7.6% |  |
|  |  | Other | 22.7% |  |
| Do you consider patients who discontinue appropriate therapy due to side effects, or who do not take appropriate therapy due to concerns about side effects, as having progressive disease? | 207 | Yes | 53.6% | No |
|  |  | No | 46.4% |  |
| Does appropriate management depend on the subtype of ILD? | 207 | Yes | 95.2% | Yes |
|  |  | No | 4.8% |  |
| If no, what constitutes appropriate management when considering progressive fibrosing ILDs as a whole?* |  | Assessing for comorbidities | 79.7% | n/a |
|  |  | Pulmonary rehabilitation | 77.6% |  |
|  |  | Trigger avoidance (e.g. smoking, antigens, occupational exposure, medications) | 77.6% |  |
|  |  | Pneumococcal vaccination and annual influenza vaccination | 72.0% |  |
|  |  | Immunomodulatory therapy | 54.6% |  |
|  |  | Genetic classification (when available) | 38.5% |  |
|  |  | Bronchoscopy with BAL | 33.6% |  |
|  |  | Watchful waiting | 31.5% |  |
|  |  | Antibiotics | 16.1% |  |
|  |  | Unsure/not applicable | 7.7% |  |
|  |  | Other (please specify) | 7.7% |  |
|  |  | Antifibrotics | 2.4% |  |
|  |  | Lung transplant | 0.5% |  |
|  |  | N/A | 2.4% |  |
| If yes, what is appropriate management in the different subtypes of ILD?* [only responses that >70% participants agreed on are shown] |  | **Assessing for comorbidities** is appropriate management for fibrotic HP | 75.4% | Yes |
|  |  | Assessing for comorbidities is appropriate management for idiopathic fibrosing NSIP | 78.5% |  |
|  |  | Assessing for comorbidities is appropriate management for IPF | 81.2% |  |
|  |  | Assessing for comorbidities is appropriate management for IPAF | 78.5% |  |
|  |  | Assessing for comorbidities is appropriate management for mixed CTD-ILD | 78.0% |  |
|  |  | Assessing for comorbidities is appropriate management for myositis-ILD | 78.5% |  |
|  |  | **Immunomodulatory therapy** is appropriate management for IPAF | 77.5% |  |
|  |  | Immunomodulatory therapy is appropriate management for mixed CTD-ILD | 89.5% |  |
|  |  | Immunomodulatory therapy is appropriate management for myositis-ILD | 89.0% |  |
|  |  | **Pneumococcal vaccination and annual influenza vaccination** are appropriate management for fibrotic HP | 88.0% |  |
|  |  | Pneumococcal vaccination and annual influenza vaccination are appropriate management for genetic ILD | 83.8% |  |
|  |  | Pneumococcal vaccination and annual influenza vaccination are appropriate management idiopathic fibrosing NSIP | 89.0% |  |
|  |  | Pneumococcal vaccination and annual influenza vaccination are appropriate management for IPF | 89.0% |  |
|  |  | Pneumococcal vaccination and annual influenza vaccination are appropriate management for IPAF | 86.4% |  |
|  |  | Pneumococcal vaccination and annual influenza vaccination are appropriate management for mixed CTD-ILD | 88.0% |  |
|  |  | Pneumococcal vaccination and annual influenza vaccination are appropriate management for myositis | 86.4% |  |
|  |  | Pneumococcal vaccination and annual influenza vaccination are appropriate management for PPFE | 82.7% |  |
|  |  | **Pulmonary rehabilitation** is appropriate management for fibrotic HP | 83.8% |  |
|  |  | Pulmonary rehabilitation is appropriate management for genetic ILD | 78.0% |  |
|  |  | Pulmonary rehabilitation is appropriate management for idiopathic fibrosing NSIP | 85.3% |  |
|  |  | Pulmonary rehabilitation is appropriate management for IPF | 86.4% |  |
|  |  | Pulmonary rehabilitation is appropriate management for IPAF | 78.5% |  |
|  |  | Pulmonary rehabilitation is appropriate management for mixed CTD-ILD | 80.6% |  |
|  |  | Pulmonary rehabilitation is appropriate management for myositis-ILd | 80.1% |  |
|  |  | Pulmonary rehabilitation is appropriate management for PPFE | 77.5% |  |
|  |  | **Trigger avoidance** is appropriate management for fibrotic HP | 91.1% |  |
|  |  | Trigger avoidance is appropriate management for idiopathic fibrosing NSIP | 74.9% |  |
|  |  | Trigger avoidance is appropriate management for IPF | 79.1% |  |
| What would trigger a change in management for your patients with ILD?* | 207 | Progression despite treatment | 96.6% | Yes |
|  |  | Worsening FVC and/or DLco | 92.3% |  |
|  |  | Starting or increasing supplemental oxygen therapy | 83.1% |  |
|  |  | Pulmonary hypertension | 77.3% |  |
|  |  | Exertional hypoxaemia | 72.0% |  |
|  |  | Unsure/not applicable | 2.4% |  |
|  |  | Other (please specify) | 3.4% |  |
|  |  | Request from patient | 0.5% |  |
|  |  | Multi-factorial assessment/changes | 0.5% |  |
|  |  | Worsened symptoms | 0.5% |  |
|  |  | Acute exacerbation | 0.5% |  |
|  |  | Progression or failure of treatment would refer to lung transplant in addition | 0.5% |  |
|  |  | Intractable cough | 0.5% |  |
|  |  | Worsening chest HRCT | 0.5% |  |

*More than one answer could be selected.

BAL, bronchoalveolar lavage; CTD-ILD, connective tissue disease-associated interstitial lung disease; DLco, diffusing capacity of the lungs for carbon monoxide; FVC, forced vital capacity; HRCT, high-resolution for computed tomography; HP, hypersensitivity pneumonitis ILD, interstitial lung disease; IPAF, interstitial pneumonia with autoimmune features; IPF, idiopathic pulmonary fibrosis; NSIP, non-specific interstitial pneumonia; PPFE, pleuroparenchymal fibroelastosis;.

**Table S7** Questions and responses on “terminology” from **A**) survey 1, **B**) survey 2 and **C**) survey 3.

**A**

| **Question/consensus statement** | **n** | **Response** | **% of respondents** | **Median and IQR for responses on Likert scale** | **Consensus?** |
| --- | --- | --- | --- | --- | --- |
| The current terminology used to describe progressive fibrosis in patients with ILD is clear and appropriate* | 207 | Strongly agree | 2.4% | Median = 1  IQR = 1 | No |
|  |  | Agree | 42.0% |  |  |
|  |  | Somewhat agree | 31.9% |  |  |
|  |  | Neutral | 7.3% |  |  |
|  |  | Somewhat disagree | 8.7% |  |  |
|  |  | Disagree | 5.3% |  |  |
|  |  | Strongly disagree | 2.4% |  |  |
| Recent clinical trials have sought to capture patients with forms of lung fibrosis that had progressed despite management. In clinical practice, what language should be used to build the definition for this condition?^†^ | 207 | Progressive fibrosing/fibrosis | 63.8% | n/a | No |
|  |  | Progressive | 49.3% |  |  |
|  |  | Progression despite therapy/management | 39.6% |  |  |
|  |  | Progressive phenotype | 32.9% |  |  |
|  |  | Progression despite immunomodulatory therapy, if indicated | 28.0% |  |  |
|  |  | Fibrosing lung disease | 27.1% |  |  |
|  |  | Fibrosing/fibrotic | 23.7% |  |  |
|  |  | Progression despite antifibrotic therapy, if indicated | 19.8% |  |  |
|  |  | Interstitial lung disease | 19.3% |  |  |
|  |  | Worsening | 15.0% |  |  |
|  |  | Other (please specify) | 4.8% |  |  |
| Do IPF and non-IPF fibrosing ILDs require different definitions of progression? | 207 | Yes | 53.1% | n/a | No |
|  |  | No | 46.9% |  |  |
|  |  | Agree | 61.7% |  |  |
|  |  | Somewhat agree | 9.6% |  |  |
|  |  | Neutral | 6.4% |  |  |
|  |  | Somewhat disagree | 3.2% |  |  |
|  |  | Disagree | 4.3% |  |  |
|  |  | Strongly disagree | 0% |  |  |

*Statements were assessed on a 7-point Likert scale. The scale was from -3 (strongly disagree/definitely not useful/not important at all) to +3 (strongly agree/definitely useful/very important). Data are % of respondents that selected each Likert scale category.

^†^More than one answer could be selected.

ILD, interstitial lung disease; IPF, idiopathic pulmonary fibrosis; PPF, progressive pulmonary fibrosis.

**B**

| **Question/consensus statement** | **n** | **Response** | **% of respondents** | **Median and IQR for responses on Likert scale** | **Consensus?** |
| --- | --- | --- | --- | --- | --- |
| Since the distribution of Survey 1, new ATS/ERS/JRS/ALAT guidelines have been published referring to PPF. According to the guidelines, PPF is defined as having at least two of three criteria (worsening symptoms, radiological progression and physiological progression) occurring within 1 year of follow-up. From your understanding, which of the following should be included in the definition of PPF? | 131 | Progressive pulmonary fibrosis within 12 months | 34.4% | n/a | No |
|  |  | Progressive pulmonary fibrosis occurring over a time interval of longer than 12 months | 7.6% |  |  |
|  |  | Both options should be part of the definition of progressive pulmonary fibrosis | 58.0% |  |  |
| PPF can be considered as: 1. Progressive pulmonary fibrosis within 12 months; 2. Progressive pulmonary fibrosis occurring over a time interval of longer than 12 months. Per the guidelines, PPF could be used to refer to one of these types of progressive pulmonary fibrosis at the exclusion of the other type. Do you find this to be a useful definition in your clinical practice for general Respirologists?* | 131 | Definitely useful | 13.7% | Median = 1  IQR = 2 | No |
|  |  | Useful | 33.6% |  |  |
|  |  | Somewhat useful | 21.4% |  |  |
|  |  | Neutral | 10.7% |  |  |
|  |  | Somewhat not useful | 9.2% |  |  |
|  |  | Not useful | 10.7% |  |  |
|  |  | Definitely not useful | 0.8% |  |  |
| Considering the term PPF as defined in the ATS/ERS/JRS/ALAT guidelines, to what extent do you agree that the terms ‘despite adequate management’ or ‘despite usual management’ should be included in the definition of PPF?* | 131 | Strongly agree | 28.2% | Median = 2  IQR = 2 | No |
|  |  | Agree | 32.8% |  |  |
|  |  | Somewhat agree | 16.8% |  |  |
|  |  | Neutral | 4.6% |  |  |
|  |  | Somewhat disagree | 7.6% |  |  |
|  |  | Disagree | 8.4% |  |  |
|  |  | Strongly disagree | 1.5% |  |  |
| Considering the term PPF as defined in the ATS/ERS/JRS/ALAT guidelines, to what extent do you find ‘despite adequate management’ or ‘despite usual management’ an important part of the definition of PPF?* | 131 | Very important | 30.5% | Median = 2  IQR = 2 | No |
|  |  | Important | 33.6% |  |  |
|  |  | Somewhat important | 14.5% |  |  |
|  |  | Neutral | 6.9% |  |  |
|  |  | Slightly not important | 2.3% |  |  |
|  |  | Not important | 10.7% |  |  |
|  |  | Not important at all | 1.5% |  |  |
| If ‘despite adequate management’ or ‘despite usual management’ is not an important part of the definition of PPF, what do you propose? | 19 | Term not needed: Define without reference to therapy or management | 15.0% | n/a | No |
|  |  | Extent of fibrosis assessed on HRCT | 1.0% |  |  |
|  |  | Other | 3.0% |  |  |
| Considering instead the phenomenon of progressive fibrosis in ILD, to what extent do you agree that the terms ‘despite adequate management’ or ‘despite usual management’ should be included in the definition of progressive fibrosis in ILD?* | 131 | Strongly agree | 26.7% | Median = 2  IQR = 2 | No |
|  |  | Agree | 32.8% |  |  |
|  |  | Somewhat agree | 17.6% |  |  |
|  |  | Neutral | 5.3% |  |  |
|  |  | Somewhat disagree | 7.6% |  |  |
|  |  | Disagree | 9.2% |  |  |
|  |  | Strongly disagree | 0.8% |  |  |
| Considering the subgroup of patients with fibrotic ILD to which antifibrotic therapy is currently applicable, please indicate to what extent you agree the term PPF is sufficient* | 131 | Strongly agree | 11.5% | Median = 2  IQR = 3 | No |
|  |  | Agree | 32.1% |  |  |
|  |  | Somewhat agree | 22.9% |  |  |
|  |  | Neutral | 8.4% |  |  |
|  |  | Somewhat disagree | 9.9% |  |  |
|  |  | Disagree | 10.7% |  |  |
|  |  | Strongly disagree | 4.6% |  |  |
| Considering the subgroup of patients with fibrotic ILD to which antifibrotic therapy is currently applicable, please indicate to what extent you agree the term PPF despite usual initial management should be specified* | 131 | Strongly agree | 29.8% | Median = 1  IQR = 2.5 | No |
|  |  | Agree | 32.8% |  |  |
|  |  | Somewhat agree | 9.2% |  |  |
|  |  | Neutral | 9.9% |  |  |
|  |  | Somewhat disagree | 6.9% |  |  |
|  |  | Disagree | 10.7% |  |  |
|  |  | Strongly disagree | 0.8% |  |  |
| To what extent do you think expert ILD clinicians would find the ATS/ERS/JRS/ALAT definition to be useful?* | 131 | Strongly agree | 32.1% | Median = 2 IQR = 2 | No |
|  |  | Agree | 33.6% |  |  |
|  |  | Somewhat agree | 21.4% |  |  |
|  |  | Neutral | 4.6% |  |  |
|  |  | Somewhat disagree | 3.1% |  |  |
|  |  | Disagree | 3.1% |  |  |
|  |  | Strongly disagree | 2.3% |  |  |
| To what extent do you think general respirologists would find the ATS/ERS/JRS/ALAT definition to be useful?* | 131 | Strongly agree | 19.9% | Median = 2  IQR = 1 | Yes |
|  |  | Agree | 39.7% |  |  |
|  |  | Somewhat agree | 28.2% |  |  |
|  |  | Neutral | 6.9% |  |  |
|  |  | Somewhat disagree | 1.5% |  |  |
|  |  | Disagree | 3.1% |  |  |
|  |  | Strongly disagree | 0.8% |  |  |
| To what extent do you think specialists in related areas would find the ATS/ERS/JRS/ALAT definition to be useful?* | 131 | Strongly agree | 21.4% | Median = 2  IQR = 1 | Yes |
|  |  | Agree | 38.2% |  |  |
|  |  | Somewhat agree | 22.1% |  |  |
|  |  | Neutral | 9.9% |  |  |
|  |  | Somewhat disagree | 1.5% |  |  |
|  |  | Disagree | 6.1% |  |  |
|  |  | Strongly disagree | 0.8% |  |  |
| To what extent do you think healthcare professionals outside speciality fields would find the ATS/ERS/JRS/ALAT definition to be useful?* | 131 | Definitely useful | 11.5% | Median = 1 IQR = 2 | No |
|  |  | Useful | 28.2% |  |  |
|  |  | Somewhat useful | 28.2% |  |  |
|  |  | Neutral | 16.8% |  |  |
|  |  | Somewhat not useful | 6.9% |  |  |
|  |  | Not useful | 7.6% |  |  |
|  |  | Definitely not useful | 0.8% |  |  |
| To what extent do you think patients would find the ATS/ERS/JRS/ALAT definition to be useful?* | 131 | Definitely useful | 13.0% | Median = 1 IQR = 2 | No |
|  |  | Useful | 33.6% |  |  |
|  |  | Somewhat useful | 26.0% |  |  |
|  |  | Neutral | 13.7% |  |  |
|  |  | Somewhat not useful | 5.3% |  |  |
|  |  | Not useful | 6.1% |  |  |
|  |  | Definitely not useful | 2.3% |  |  |
| To what extent do you think policy makers/payers would find the ATS/ERS/JRS/ALAT definition to be useful?* | 131 | Definitely useful | 21.4% | Median = 2  IQR = 1 | Yes |
|  |  | Useful | 38.9% |  |  |
|  |  | Somewhat useful | 21.4% |  |  |
|  |  | Neutral | 10.7% |  |  |
|  |  | Somewhat not useful | 3.8% |  |  |
|  |  | Not useful | 2.3% |  |  |
|  |  | Definitely not useful | 1.5% |  |  |

*Statements were assessed on a 7-point Likert scale. The scale was from -3 (strongly disagree/definitely not useful/not important at all) to +3 (strongly agree/definitely useful/very important). Data are % of respondents that selected each Likert scale category.

^†^More than one answer could be selected.

ILD, interstitial lung disease; IPF, idiopathic pulmonary fibrosis; PPF, progressive pulmonary fibrosis.

**C**

| **Question/consensus statement** | **n** | **Response** | **% of respondents** | **Median and IQR for responses on Likert scale** | **Consensus?** |
| --- | --- | --- | --- | --- | --- |
| To what extent do you agree with the following: “Progression occurring either within 12 months or over a longer time interval, should both be considered as progressive pulmonary fibrosis”?* | 94 | Strongly agree | 30.9% | Median = 2  IQR = 2 | No |
|  |  | Agree | 41.5% |  |  |
|  |  | Somewhat agree | 12.8% |  |  |
|  |  | Neutral | 2.1% |  |  |
|  |  | Somewhat disagree | 6.4% |  |  |
|  |  | Disagree | 5.3% |  |  |
|  |  | Strongly disagree | 1.1% |  |  |
| To what extent do you agree with the following: “Considering either the term PPF as defined in the ATS/ERS/JRS/ALAT guidelines or the phenomenon of progressive fibrosis in ILD, the terms ‘despite adequate management’ or ‘despite usual management’ are important and should be included in the definition of progression”?* | 94 | Strongly agree | 30.9% | Median = 2  IQR = 1 | Yes |
|  |  | Agree | 50.0% |  |  |
|  |  | Somewhat agree | 7.5% |  |  |
|  |  | Neutral | 6.4% |  |  |
|  |  | Somewhat disagree | 0% |  |  |
|  |  | Disagree | 5.3% |  |  |
|  |  | Strongly disagree | 0% |  |  |
| To what extent do you agree with the following: “Considering the subgroup of patients with fibrotic ILD to which antifibrotic therapy is currently available as an option, PPF ‘despite usual initial management’ would be a useful part of the definition”?* | 94 | Strongly agree | 14.9% | Median = 2  IQR = 0 | Yes |
|  |  | Agree | 61.7% |  |  |
|  |  | Somewhat agree | 9.6% |  |  |
|  |  | Neutral | 6.4% |  |  |
|  |  | Somewhat disagree | 3.2% |  |  |
|  |  | Disagree | 4.3% |  |  |
|  |  | Strongly disagree | 0% |  |  |

*Statements were assessed on a 7-point Likert scale. The scale was from -3 (strongly disagree/definitely not useful/not important at all) to +3 (strongly agree/definitely useful/very important). Data are % of respondents that selected each Likert scale category.

^†^More than one answer could be selected.

ILD, interstitial lung disease; IPF, idiopathic pulmonary fibrosis; PPF, progressive pulmonary fibrosis.
